# Aluminum Micropillar Surfaces with Hierarchical Micro- and Nanoscale Features for Enhancement of Boiling Heat Transfer Coefficient and Critical Heat Flux

**DOI:** 10.3390/nano14080667

**Published:** 2024-04-11

**Authors:** Armin Hadžić, Matic Može, Matevž Zupančič, Iztok Golobič

**Affiliations:** Faculty of Mechanical Engineering, University of Ljubljana, Aškerčeva 6, 1000 Ljubljana, Slovenia; armin.hadzic@fs.uni-lj.si (A.H.); matevz.zupancic@fs.uni-lj.si (M.Z.); iztok.golobic@fs.uni-lj.si (I.G.)

**Keywords:** nucleate boiling, heat transfer enhancement, critical heat flux, heat transfer coefficient, micro-nanoscale structures, micropillar porous surfaces, nanosecond laser texturing

## Abstract

The rapid progress of electronic devices has necessitated efficient heat dissipation within boiling cooling systems, underscoring the need for improvements in boiling heat transfer coefficient (HTC) and critical heat flux (CHF). While different approaches for micropillar fabrication on copper or silicon substrates have been developed and have shown significant boiling performance improvements, such enhancement approaches on aluminum surfaces are not broadly investigated, despite their industrial applicability. This study introduces a scalable approach to engineering hierarchical micro-nano structures on aluminum surfaces, aiming to simultaneously increase HTC and CHF. One set of samples was produced using a combination of nanosecond laser texturing and chemical etching in hydrochloric acid, while another set underwent an additional laser texturing step. Three distinct micropillar patterns were tested under saturated pool boiling conditions using water at atmospheric pressure. Our findings reveal that microcavities created atop pillars successfully facilitate nucleation and micropillars representing nucleation site areas on a microscale, leading to an enhanced HTC up to 242 kW m^−2^ K^−1^. At the same time, the combination of the surrounding hydrophilic porous area enables increased wicking and pillar patterning, defining the vapor–liquid pathways on a macroscale, which leads to an increase in CHF of up to 2609 kW m^−2^.

## 1. Introduction

Nucleate pool boiling, a very efficient heat dissipation method, is commonly employed for cooling energy-intense applications such as power electronics [1,2], superconducting magnets [3,4], and nuclear power plants [5,6]. Pool boiling represents one of the most intense cooling methods, alongside spray cooling, microchannel heat sinks, and heat removal techniques involving porous media [7,8,9]. Efficient heat transfer during boiling is the result of latent heat consumed by the liquid–vapor phase change. The major parameters to quantify the efficacy of the nucleate boiling process are the critical heat flux (CHF) and the heat transfer coefficient (HTC). The latter is defined as the ratio of heat flux to the wall superheat (Δ*T*_w_). Here, Δ*T*_w_ is the temperature difference between the boiling surface and the liquid. The critical heat flux signifies the upper limit of the nucleate boiling regime, manifesting when an excessive generation of vapor bubbles impedes the rewetting of the surface, thus causing the formation of an insulating vapor film over the surface. This vapor film acts as a thermal barrier, leading to a pronounced rise in wall superheat, which frequently results in severe damage to the system. Hence, the pursuit of the improved safety of boiling systems, coupled with the continuous increase in heat dissipation requirements, underscores the need for further enhancements of the critical heat flux and the overall boiling performance.

Efforts directed toward the enhancement of CHF and HTC have primarily been focused on the modification of the properties of the working fluid or the boiling interface. The engineering of surface structures is receiving more attention, primarily due to limitations of chemical compatibility or operational requirements, which often limit the selection of the working fluid. In past decades, researchers have proposed various methodologies for the engineering of boiling surfaces to effectively enhance CHF and HTC. The main surface engineering techniques include milling, drilling, electrochemical etching, chemical etching, laser processing, and sintering, among others. For example, surfaces with engineered structures that have demonstrated CHF and HTC improvement include (i) microstructures, comprised of elements like fins [10,11], pillars [12,13], microcavities [14,15], and pores; (ii) nanostructures, made of nano-porous media [16,17], nanotubes [18,19], nanowires [20,21], and nano-coatings [22,23]; and (iii) micro/nano hierarchical structures [24,25].

A surface with engineered micro/nanostructures can exhibit an extended active heat transfer area, leading to an increase in the nucleation site density during boiling [26,27]. Additionally, micro/nano wicking structures characterized by superhydrophilicity lead to an intensified capillary force, which results in increased liquid supply and the mitigation of dry spot formation [28,29]. Evidently, both the CHF and the HTC on structured surfaces are significantly influenced by surface wettability. The latter plays a major role in bubble dynamics by governing processes such as nucleation, growth, coalescence, and departure [30]. Increased surface wettability leads to a delayed CHF incipience, which can be attributed to more intense liquid rewetting, while decreased surface wettability results in promoted bubble formation due to a reduced nucleation energy barrier [31,32,33]. The synergy between increased bubble nucleation sites and enhanced liquid replenishment serves to further facilitate boiling heat transfer, a concept that has been advanced by the design of surfaces with locally mixed wettability [15,34,35].

By simultaneously combining the aforementioned mechanisms aimed at enhancing CHF and HTC, several investigations have reported notable boiling heat transfer performance improvements on micro-nanotextured surfaces, utilizing smooth surfaces as the benchmark. Song et al. [36], who utilized micropillar surfaces, demonstrated CHF values up to 2600 kW m^−2^, while the highest recorded HTC values were up to 100 kW m^−2^ K^−1^. The CHF enhancement was attributed to the minimization of bubble coalescence and an increase in capillary wicking by separating nucleation sites using tube clusters. Liu et al. [37] investigated the boiling heat transfer performance under subcooled conditions on silicon micro-pin-fin surfaces employing FC 72 as the working fluid. The findings of this study revealed that surfaces featuring nanostructures etched at the top edges of micro-pin-fins exhibited the highest values for both CHF and HTC. This phenomenon is attributed to the predominant bubble generation occurring primarily on the tops of micro-pin-fins, thereby mitigating the blockage of the capillary wicking channels by the bubbles in the high heat flux region. Lee et al. [38] investigated pool boiling performance utilizing deionized water on structured silicon surfaces under saturated conditions. Their results revealed that surfaces featuring a hybrid micro-nanostructure exhibited the highest CHF, reaching up to 2600 kW m^−2^. This achievement is attributed to the delay of the bubble merger and the maximization of bubble density. Shim et al. [39] designed silicon surfaces featuring superbiphilic patterned nanowires and investigated pool boiling performance using deionized water under saturated conditions. The results demonstrated a significant increase in the CHF value, up to 100%, in comparison to the plain surface. This enhancement was attributed to the wicking characteristics of the superhydrophilic region and the facilitation of vapor–liquid pathways through bubble separation. Furthermore, they also observed an enhancement in HTC attributed to promoting nucleation and maximizing nucleate site density. On the other hand, HTC enhancement was ascribed to nucleation promoted by microscale cavities. Wang et al. [40] developed multiscale aluminum surfaces to study the influence of various wetting states on boiling performance. They showed improvement in CHF and HTC by 162.1% and 180.8% compared to the flat sample, respectively. Superhydrophilic multiscale structures enabled enhanced liquid replenishment of the drying area, while pyramid structures hindered the expansion of vapor films, resulting in increased CHF. The improvement in HTC was attributed to improved nucleation due to the fabricated micro-nanotextured surfaces. Kim et al. [41] evaluated boiling performance on well-organized micropillar structured silicon surfaces, demonstrating an HTC up to 30% higher over the reference surface, with the highest recorded CHF reaching up to 2100 kW m^−2^. Song et al. [42] designed precisely controlled microtube structures, where a cavity was defined at the center of a pillar, to simultaneously enhance HTC and CHF. Cavities trapped vapor and improved bubble nucleation to enhance HTC, while the sidewalls of the microtube arrays provided CHF enhancement by increasing capillary wicking. Cho et al. [43] reported the enhancement of CHF and boiling performance on silicon micro-nanotextured surfaces with mixed wettability. The CHF was improved up to 1900 kW m^−2^ by the fabricated nanostructure that enables better rewetting of the dried area, while improved boiling performance was achieved by imparting hydrophobicity at the top of the micropillars to increase the nucleation site density. Sun et al. [44] demonstrated enhanced CHF and HTC values on micropillar copper surfaces with mixed wettability. Specifically, the highest HTC and CHF were 257.6 kW m^−2^ K^−1^ and 2190.8 kW m^−2^, respectively. They found that the area of the hydrophobic region plays an important role in achieving high HTC by increasing nucleation site density and promoting bubble coalescence, while the microstructure promotes liquid supply in the high heat flux region to delay the CHF. It is noteworthy that prior investigations involving micropillars predominantly focused on silicon and copper substrates, with minimal attention directed toward aluminum surfaces. Aluminum is low-cost with lower density compared to copper, which is often used in heat transfer engineering. The thermal conductivity of aluminum is high enough, warranting the development of structured surfaces for enhanced boiling performance. Some previous studies already focused on developing aluminum-structured surfaces, albeit without micropillars, for improving boiling performance. For example, Može et al. [14] showed extreme boiling performance improvements when utilizing superhydrophobic laser-textured aluminum surfaces. The extreme HTC enhancement was attributed to the existence of microcavities that promote nucleation, especially in a hydrophobic state. A notable CHF enhancement was also recorded on superhydrophobic surfaces which was ascribed to the laser-fabricated structure. Godinez et al. [45] developed nanoporous aluminum surfaces utilizing hot water treatment. The highest recorded CHF and HTC values were 1850 kW m^−2^ and 59 kW m^−2^ K^−1^, respectively. The CHF enhancement was achieved with improved liquid supply by the fabricated nanoporous layer, formed during the hot water treatment. Može et al. [34] also investigated laser-textured aluminum surfaces with mixed wettability and showed an increase in HTC and CHF, achieving greatly enhanced heat transfer coefficients at high heat fluxes. This was attributed to the increased density of potential active nucleation spots, which was especially pronounced on surfaces with a superhydrophobic area fraction of 23%. Evidently, few studies investigated the improvement in boiling performance on structured aluminum surfaces, achieving some noteworthy enhancements of either HTC or CHF. However, numerous studies have been dedicated to the development of hierarchical micro/nano-scale structures on copper and silicon substrates, with the objective of enhancing the CHF and HTC during boiling processes [46,47,48,49]. In addition, a limited number of studies listed above have attempted to develop hierarchical surfaces with micro/nano-scale features on aluminum substrates, aiming to achieve simultaneous enhancement of CHF and HTC during pool boiling.

### Motivation and Goals of the Present Study

The preset study aims to investigate the possibility of significant HTC and CHF enhancements by developing a hybrid micro-/nanostructured surface utilizing the combination of direct laser texturing and chemical etching on aluminum samples. Despite the valuable contributions of previous investigations, the exploration of boiling enhancement methods on aluminum surfaces has not been extensively pursued, and there exists a notable lack of knowledge in the approaches directed toward the development of scalable hierarchical micro-/nanoscale features on aluminum substrates to concurrently enhance CHF and HTC during pool boiling.

This research outlines the development of a scalable approach for engineering hierarchical surface structures on aluminum substrates, capable of incorporating microcavities for vapor entrapment to facilitate nucleation, micropillars to extend the surface area and provide wicking capabilities, an intermediate hydrophilic porous region to further enhance wicking, and pillar patterning to define vapor–liquid pathways on the macroscale with the main aim of enhancing pool boiling heat transfer and critical heat flux. One set of micropillar aluminum surfaces was fabricated utilizing nanosecond laser texturing and chemical etching in 3M hydrochloric acid, while the second set of micropillar surfaces underwent an additional laser texturing step after the chemical etching. The boiling heat transfer performance was evaluated by pool boiling tests with water, conducted under saturated conditions at atmospheric pressure. The impact of hierarchical micro- and nanoscale surface structures, as well as micropillar height and patterning, on the boiling performance enhancement was evaluated by utilizing two different heights of micropillars, and three different micropillar pattern enhancements were evaluated by utilizing two different heights of micropillars and three different micropillar patterns.

## 2. Materials and Methods

### 2.1. Samples

The samples were fabricated from 6082 aluminum alloy rods. The upper flat face of these samples (with a diameter of 14 mm) served as the boiling interface, while the lower flat face of the sample was mounted onto the heating block. The boiling surface of each sample underwent a sanding process utilizing P1200 and P2000 sandpaper, resulting in an approximate surface roughness value of *R*_a_ = 0.20 μm. The surface roughness of the tested samples was measured using a Mitutoyo SJ-310 device (Mitutoyo Corporation, Kanagawa, Japan). Samples were cleaned using 2-propanol before testing or any following treatments. To obtain the axial temperature gradient within the sample, calculate the heat flux, and extrapolate the surface temperature, three K-type thermocouples were glued into each sample. A schematic view of the sample with corresponding dimensions is shown in Figure S1 in the Supporting Information, Section S1.

### 2.2. Fabrication of Micropillar Surfaces

**Figure 1:**
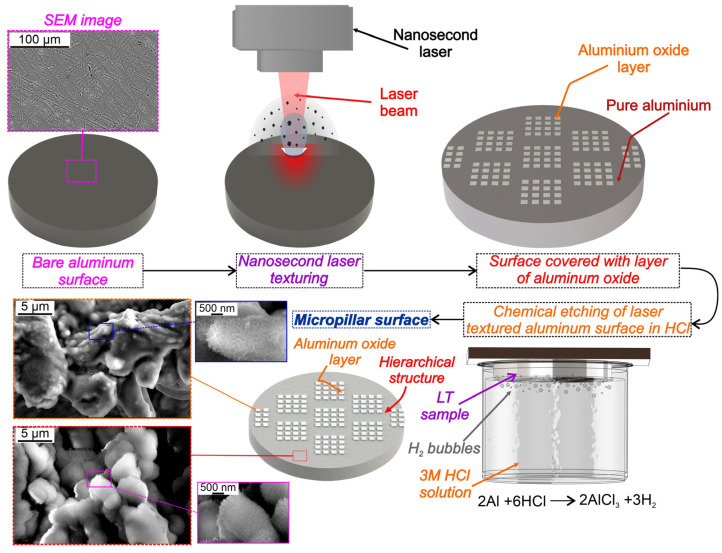
Fabrication process of micropillar aluminum surfaces.

Micropillars were engineered on aluminum surfaces through a two-step approach involving nanosecond laser texturing and chemical etching. The laser texturing process was performed with a nanosecond fiber laser (JPT Opto-electronics Co., Ltd., Shenzhen, China, M7 30 W MOPA; *λ* = 1064 nm). The laser beam employing an F-Theta lens with a 70 × 70 mm^2^ working area and a focal distance of 100 mm was directed across the surface. The focused beam featured an approximate diameter of 25 μm, with a manufacturer-reported laser beam quality of M^2^ < 1.3, and a maximum laser source power of 30 W. The design of surface treatment patterns was achieved with the “ezCAD2” software (version ezCAD2.14.11), which was also used to control the laser system during treatment. The entire micropillar fabrication process on aluminum samples is shown in Figure 1.

In the first fabrication step, square patches of 400 × 400 μm were created using nanosecond laser texturing, serving as a base for further preparation of micropillars. These squares were composed of parallel grooves and ridges consisting of porous resolidified material. During the laser texturing process, thin layers of aluminum oxide formed atop the squares. This procedure also produced microcavities, which were previously shown to be remarkably effective as preferential nucleation sites during nucleate boiling [14,15]. Laser texturing parameters used for the fabrication of square patches, covered by aluminum oxides, were as follows: scanning speed of 110 mm s^−1^, pulse frequency of 110 kHz, pulse width of 45 ns, average power of 30 W, and average pulse fluence of 55.6 J cm^−2^. The scanning pattern was configured with parallel lines featuring cyclically variable spacing oscillating by 5 μm within the range of 75 μm to 85 μm. The laser texturing used for the fabrication of square patches, serving as foundations for micropillar creation, is graphically shown in Figure S2 in the Supporting Information, Section S2.

The subsequent step of micropillar fabrication consisted of chemical etching on the samples with laser-textured square base patterns. This procedure was employed to increase the height of the micropillars after the initial laser texturing step by predominantly etching away the material between textured areas. A 3M solution of hydrochloric acid (HCl) was utilized for the etching process. The entire surface was immersed in the acid solution to initiate a reaction between HCl, aluminum oxide, and the surrounding pure aluminum. The reaction between aluminum oxide and HCl is much slower compared to the one involving pure aluminum and HCl [50,51]. This difference is attributed to the protective influence of the Al_2_O_3_ layer, a byproduct of laser texturing, that covers the irradiated squares and impedes the contact between HCl and the underlying aluminum [50,51]. Hence, the overall reaction rate is greatly diminished compared to the reaction rate on areas consisting of pure aluminum. After the procedure, this leads to an increased depth of areas that surround the squares and thus the formation of micropillars. To achieve different micropillar heights, the number of laser passes (five and ten passes) and the etching time (20 and 40 min) were varied. Surfaces textured through five laser passes underwent a 20 min immersion in HCl, while those with ten laser passes were immersed for 40 min. Longer etching led to a more pronounced reaction between HCl and pure aluminum, which also began to appear underneath the aluminum oxide layer, rendering the pillars unstable. 

**Figure 2:**
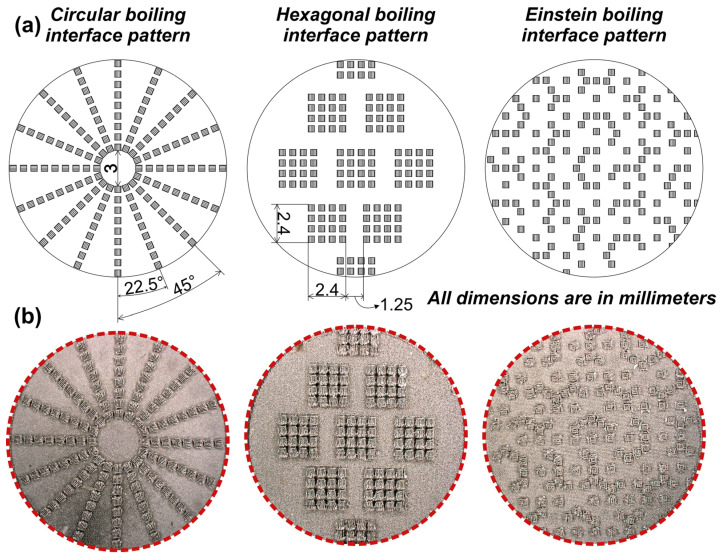
Boiling interfaces fabricated on aluminum surface: (**a**) graphical representation with corresponding dimensions and (**b**) macroscopic surface images after preparation.

Three micropillar patterns were fabricated on surfaces, i.e., circular, hexagonal, and Einstein patterns. The circular pattern was chosen for its ability to possibly segregate vapor and liquid supply, which leads to an increase in CHF [52]. The hexagonal pattern was engineered to observe the impact of groups of micropillars in all directions on both CHF and HTC [15,34]. The Einstein pattern was designed to investigate how the non-repetitive spatial distribution of nucleation-promoting areas influences critical heat flux and boiling heat transfer coefficient [53,54]. This distinct 13-sided tile pattern, characterized by its non-repetitive configuration, was generated through the formation of micropillars along the edges of said tiles. The micropillar patterns fabricated in this study are shown in Figure 2.

### 2.3. Fabrication of Fully Laser-Functionalized Micropillar Surfaces

The second set of micropillar surfaces was fabricated by utilizing the aforementioned preparation method, combined with an additional laser texturing step. The second step of laser texturing with the same parameters as before was employed on the surrounding area of the micropillar, creating grooves and ridges by ablation, melting, and subsequent resolidification. This added fabrication step was used to produce multiscale porous structures on the micropillar surrounding areas after chemical etching and is graphically explained in Figure S3 in the Supporting Information, Section S2.

### 2.4. Pool Boiling Heat Transfer Evaluation

Pool boiling experiments at atmospheric pressure were conducted using the experimental setup shown in Figure S4, Supporting Information, Section S3. The glass boiling chamber with an inner diameter of 60 mm was filled with 200 mL of double distilled water. The saturation temperature was achieved and maintained with an immersion heater, while the sample was heated with three cartridge heaters inside the heating block. The water temperature was measured with two submerged K-type thermocouples at different heights inside the chamber. A condenser, cooled by a secondary water loop, was mounted on top of the assembly to keep the water level steady during the experiments. All temperature signals were recorded as raw voltages at 10 Hz, using a DAQ device (KRYPTIONi-8xTH; Dewesoft, Trbovlje, Slovenia) and Dewesoft X3 2022.1 software. Additional information about the experimental setup is available in our previous work [15,31]. 

The measurement protocol in this study employed the degassing of water in the boiling chamber to ensure a pure boiling liquid free of non-condensable gases. This was accomplished by first activating the immersion heater for 30 min and then switching on the cartridge heaters for an additional 15 min to maintain boiling at an approximate intensity of 250 kW m^−2^ s^−1^. This procedure enabled the creation of a degassed surface free of trapped vapor or gas, effectively preventing the early onset of nucleate boiling. Following the degassing phase, the temperature was lowered to below 90 °C to allow any remaining vapor pockets to condense. Thereafter, measurements were conducted under atmospheric pressure and at saturation temperature. In the process of recording a boiling curve, the heat flux increment was maintained at less than 0.2 kW m ^−2^ s^−1^ during the natural convection phase and under 2 kW m ^−2^ s^−1^ during the nucleate boiling phase. This dynamic approach to measurement was previously assessed and validated in the study [14]. For some samples, only one experimental run was performed due to the high heat fluxes, resulting in high surface temperatures that caused either the adhesive or the PEEK bushing to melt.

### 2.5. Data Reduction and Measurement Uncertainty

Altogether, five temperatures were measured during the experiment—three inside the sample and two in the water. From these, the heat flux, surface superheat, and heat transfer coefficient were calculated and recorded in real time with the Dewesoft X3 2022.1 software, while the post-processing was conducted with Mathworks MATLAB R2021a. Additional information is available in our previous works [31,32].

Briefly, temperature-dependent thermal conductivity was calculated at the average sample temperature, obtained as the arithmetic mean of three thermocouple measurements. Heat flux was calculated with the Fourier law of one-dimensional heat conduction under the assumption of steady-state conditions using the temperature gradient obtained from the thermocouple measurements. Surface superheat was obtained as the difference between the sample surface temperature and water temperature. The latter was taken as the arithmetic mean of both submerged thermocouple measurements, while the surface temperature was extrapolated from the temperature measurement closest to the surface and the heat flux. The heat transfer coefficient was obtained by dividing the heat flux with the surface superheat.

The evaluation of standard measurement uncertainty was conducted by the following recommendations from a previous study [15,34]. The values for the three crucial heat transfer parameters are presented in Table 1. 

All evaluations of boiling performance were conducted using the dynamic boiling curve measurement method, consistent with our previous studies, which were proven to yield accurate results for heat transfer parameters and significantly expedite the measurement process [55]. This approach offers the advantage of reducing the potential impact of surface property changes on the measurement results, while also enabling the acquisition of multiple boiling curves to assess the stability of the surface and its heat transfer performance.

## 3. Results

### 3.1. Surface Analysis of Micropillar Surfaces

Two distinct sets of aluminum micropillar surfaces were prepared through chemical etching in hydrochloric acid (HCl) and laser texturing. The first set of surfaces only underwent laser texturing before etching in HCl, while the second set of surfaces received a second laser treatment after the HCl etching.

**Figure 3:**
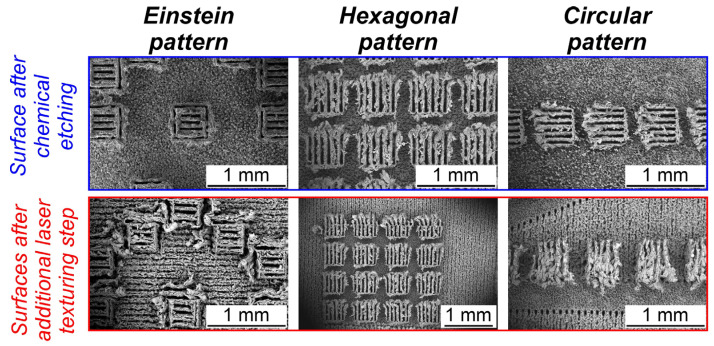
SEM images: micropillar sample after chemical etching (**top**) and micropillar samples after additional laser texturing step (**bottom**).

Depending on the chemical etching time, micropillars with different heights were produced. Furthermore, surface structures in the form of ˝micropeaks˝ were produced on the pure aluminum surrounding areas during chemical etching. These porous structures are suitable for providing increased capillary wicking [55,56]. The contact angle recorded prior to boiling on this part of the surface was less than 5°. Previous literature demonstrated that superhydrophilicity and porosity generally enhance boiling performance, particularly the critical heat flux values [23,57]. To analyze the aforementioned surface micro- and nanostructures, a scanning electron microscope (SEM; ThermoFisher Scientific Quattro S, Waltham, MA, USA) was used. SEM images of the first set of fabricated micropillar aluminum surfaces are shown in Figure 3 (top row).

The fabrication of the second set of micropillar aluminum surfaces involved an additional laser texturing step following chemical etching. SEM images of the second set of surfaces are shown in Figure 3 (bottom row). Additional SEM images of the tested surfaces are shown in Figures S5–S10 in the Supporting Information, Section S4.

**Figure 4:**
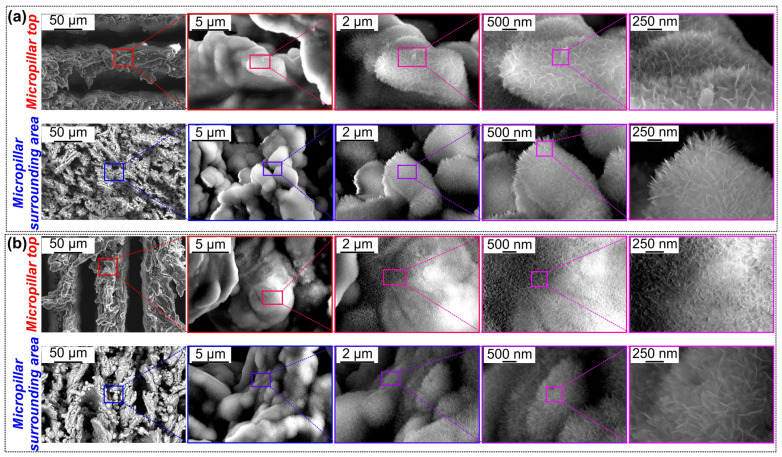
(**a**) SEM images after boiling test on micropillar sample after chemical etching: top of the micropillar (**top**) and micropillar surrounding area (**bottom**). (**b**) SEM images after boiling test on micropillar sample with additional laser texturing step: top of the micropillar (**top**) and micropillar surrounding area (**bottom**).

Figure 4 shows SEM images of the micro-nanostructured samples after the boiling test (sample tested immediately after chemical etching, see Figure 4a, and sample tested after an additional laser texturing step, see Figure 4b). At the microscale, it was observed that after boiling tests, there was a noticeable difference between the aforementioned samples. On the other hand, there is no clear difference between the tested samples after boiling at the nanoscale. The samples were exposed to boiling in hot water for several hours, which led to the formulation of the aluminum oxide hydroxide, i.e., boehmite nanoneedle structures on surfaces. Furthermore, the boehmite nanoneedles are observed both on the top of the micropillar and in the surroundings of the micropillar on both sets of tested surfaces. The observation of the boehmite nanoneedles on surfaces after the exposure of aluminum samples to boiling water several times is in agreement with previous findings [45,55].

The collection of surfaces prepared and tested within the study is summarized in Table 2.

### 3.2. Pool Boiling Performance of Micropillar Surfaces

**Figure 5:**
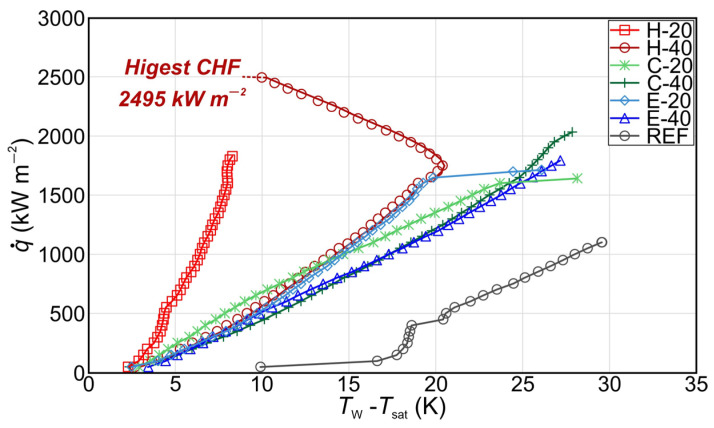
Boiling curves recorded on micropillar surfaces.

Pool boiling experiments on fabricated aluminum micropillar surfaces were conducted at atmospheric pressure using double distilled water. Boiling curves, recorded up to CHF incipience, are shown in Figure 5 for all tested samples. As a benchmark for enhancement comparison, the bare aluminum surface was denoted as reference (REF). In Figure 5, the boiling curves recorded during the first experimental run are presented. For surfaces characterized by exceedingly high CHF values, it was not possible to carry out a subsequent boiling run because of the very high temperatures (i.e., above 250 °C) reached at the bottom of the aluminum sample and the consequent destruction of the PEEK flange. The CHF obtained on the reference surface was 1100 kW m^−2^. The highest CHF of 2495 kW m^−2^ was recorded on the sample with a hexagonal pattern of higher micropillars, representing an enhancement of 126% over the reference surface. All other tested micropillar samples also exhibited a CHF enhancement over the reference surface within the range of 48% to 93%. Moreover, on sample H-40, the boiling curve “hook back” effect was observed. This can be attributed to the activation of the additional nucleation sites on the top of the laser microstructures in the region of high heat fluxes, as was established by Kruse et al. [58] and observed in other studies as well [59,60].

**Figure 6:**
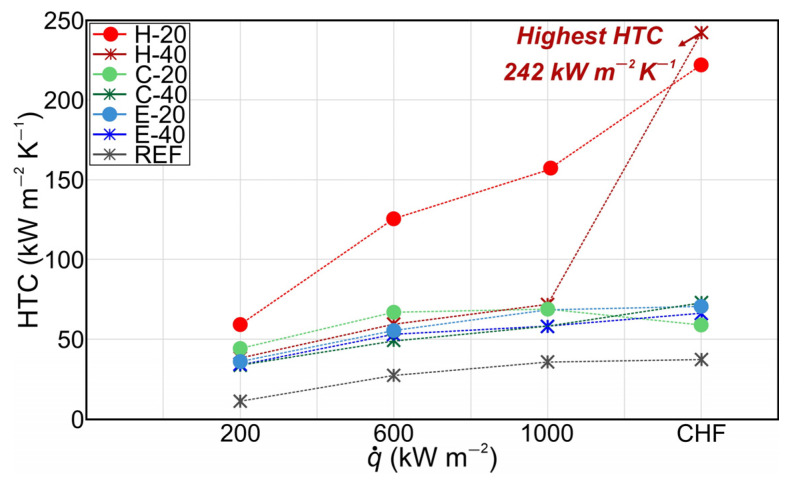
Heat transfer coefficients recorded on micropillar surfaces.

Figure 6 shows the HTC values of tested surfaces over different heat flux levels. The highest HTC of 242 kW m^−2^ K^−1^ was recorded on the surface with a hexagonal pattern of higher micropillars at CHF with a relative enhancement of 554% over the reference sample. All other tested surfaces exhibited the highest HTC at CHF with a relative enhancement over the benchmark surface within the range of 57% to 560%.

Generally, CHF enhancement can be attributed to increased capillary wicking and better liquid rewetting of the surface, while HTC enhancement can be attributed to the fabricated microcavities that serve as potential active nucleation sites and can increase the nucleation site density. This is in agreement with previous research where applying microcavities on the surface due to laser texturing ensured enhanced boiling performance [14,15]. Furthermore, the noteworthy improvement in boiling heat transfer parameters observed on surfaces with micropillars may be attributed to the influence of the formation of an intra-pillar liquid layer. The nucleation of bubbles atop the pillars leads to the creation of the intra-pillar liquid layer that can play an important role in preventing dry spot formation, therefore leading to a significant enhancement of CHF, as shown by Wang et al. [61]. Therefore, it can be concluded that the observed CHF enhancement on the micropillar surfaces is not just a result of enhanced liquid wicking but also of the trapped liquid in the intra-pillar layer, which plays a significant role in the improvement in CHF [61,62,63].

**Figure 7:**
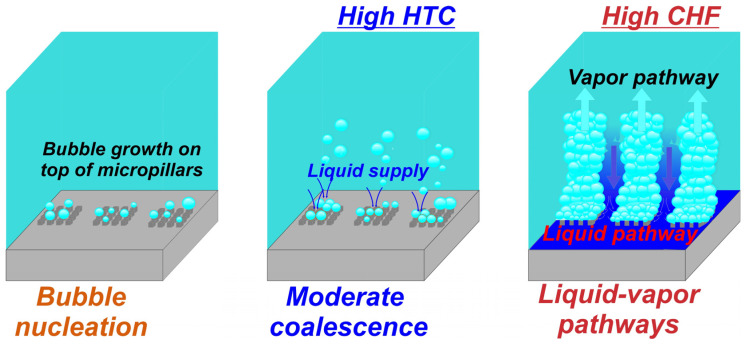
Bubble interactions on the best-performing surface pattern.

Furthermore, the results indicate a significant role of the micropillar pattern in the simultaneous enhancement of boiling parameters. One can observe that the hexagonal pattern of micropillars demonstrated the highest simultaneous enhancement compared to the two other patterns at both micropillar heights. A possible reason for this is the interplay of neighboring bubbles, which form on top of the micropillars and coalesce horizontally with each other, creating vapor patches on each group of micropillars, leaving the liquid pathways intact. This behavior enables improved rewetting of the bottom part of the surface, which helps to distribute the liquid over the surface, avoiding dryouts and the formation of vapor film, which leads to the early transition into film boiling. On the other hand, the interaction of bubbles on surfaces with circular patterns is insufficient, which results in the creation of vapor patches at the center of the pattern, hindering liquid supply and resulting in earlier CHF incipience compared to hexagonal patterns. The bubbles on non-repetitive distributed micropillars (Einstein pattern) interact with each other at the very low micropillar pitch on some parts of the sample which is highly suitable for the formation of small vapor films on such parts of the sample, causing eventual merging and possible earlier incipience of the boiling crisis. The bubble interactions on the surface with the hexagonal boiling interface pattern (the best-performing surface) are graphically shown in Figure 7.

Furthermore, it is observed that the augmented surface area also influences the enhancement of the boiling performance observed on the micropillar surface. The surface area increased on treated surfaces due to the presence of micropillars is analyzed in Table S1 in the Supporting Information, Section S5. Boiling curves at different heat flux levels, calculated using the augmented surface area, are shown in Figure S11 in the Supporting Information, Section S5. It is observed that the highest CHF values are obtained on the surfaces with hexagonal patterns, while the lowest CHF values were recorded on surfaces with the Einstein pattern, utilizing the actual surface area for heat flux calculation. The observed differences in CHF values between micropillar-patterned surfaces, i.e., hexagonal, circular, and Einstein pattern samples, can be attributed to the distinct underlying boiling mechanisms operating on these surfaces, rather than being solely influenced by the augmentation of surface area.

Moreover, for surfaces where the glue and PEEK bushing did not melt after the first experimental run, we performed three experimental runs to obtain the stability of the boiling performance on those surfaces. On the other hand, it was not possible to carry out a second experimental run on the H-40 sample. Therefore, to ensure the reliability of the fabrication process and measurements, we fabricated another H-40 surface using the same fabrication procedure and conducted the boiling experiments. Figure S13 shows the stability of the boiling performance recorded on surfaces with smaller micropillars, while the stability of the boiling performance recorded on surfaces with taller micropillars is shown in Figure S14, Supporting Information, Section S6.

### 3.3. Pool Boiling Performance of Micropillar Surfaces Treated with Second Laser Texturing Step

After the initial study, the boiling performance of micropillar surfaces was additionally increased using additional laser functionalization of the bottom part of the boiling surface.

**Figure 8:**
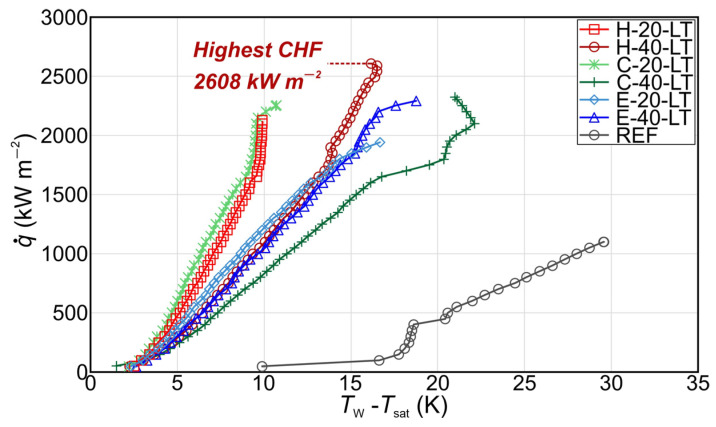
Boiling curves recorded on micropillar surfaces treated with a second laser texturing step.

Boiling curves recorded during the first experimental run of micropillar surfaces with the additional step of laser functionalization are shown in Figure 8. The results revealed that surfaces with a hexagonal pattern and higher micropillars demonstrated the highest CHF of 2608 kW m^−2^, with enhancement over the bare aluminum surface of 137%.

Surfaces with the Einstein boiling interface pattern demonstrated the lowest enhancement of CHF values, which can be attributed to the insufficient interaction between the bubbles, especially at high heat flux levels where nucleation site density is increased. This higher number of bubbles and low pitch between micropillars can result in the formation of vapor-induced resistance to liquid flow, leading to the creation of dryouts over different parts of the surface and earlier incipience of the CHF. Moreover, an important role in the interaction between the bubbles on the Einstein pattern is also the distance between the micropillars. A smaller spacing between some micropillars in certain regions of the sample with the Einstein pattern contributed to an excessive coalescence of bubbles, which led to the formation of vapor patches in those regions and reduced liquid supply, resulting in a lower enhancement of boiling performance. Furthermore, the circular pattern of the micropillars distributed in lines (see Figure 2) exhibited higher CHF values compared to the non-repetitive distribution of micropillars (the Einstein pattern). The circular pattern has a larger laser-textured area between the lines of micropillars, which prevents excessive lateral coalescence between bubbles to some extent and increases liquid supply, leading to a delay of transition toward film boiling. However, on the surface with the hexagonal patterns, the micropillars grouped into a square matrix of 4 × 4 pillars showed the highest CHF of all tested surfaces. The laser-induced structure between the groups of micropillars enables the highly efficient liquid supply to the micropillars, preventing the formation of a vapor film. On the other hand, the moderate lateral coalescence of bubbles formed on micropillars in each group was observed. The lateral coalescence of the bubbles in a micropillar group led to the formation of smaller vapor patches over each micropillar group, creating distinct vapor pathways on the surface. Thus, the surface with a hexagonal pattern (groups of micropillars with a square matrix of 4 × 4 pillars), due to the specific distribution of micropillars, enabled the formation of distinct vapor–liquid pathways that led to the significant CHF enhancement. 

The boiling curves using the actual surface area in the heat flux calculation, as opposed to the projected area of the heater for aluminum micropillar surfaces (after an additional laser texturing step), are shown in Figure S12 in the Supporting Information, Section S5. It can be observed that in this case, too, the augmentation of the surface has an impact on CHF enhancement. However, the most effective surface regarding CHF was a surface with a hexagonal pattern and higher micropillars among those tested within this group, which agrees with the observations made when comparing boiling curves utilizing the projected surface area for heat flux calculation. Hence, similarly, as with surfaces that were tested immediately after chemical etching, the CHF enhancement cannot be solely attributed to the increased surface area, but the role of the micropillar pattern and underlying mechanisms has a significant influence on CHF enhancement.

**Figure 9:**
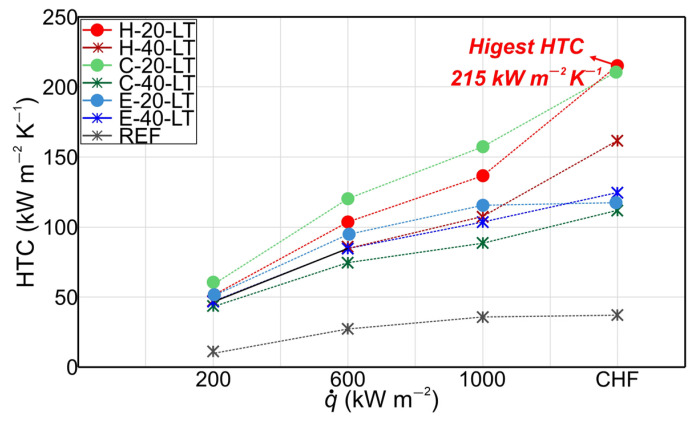
Heat transfer coefficients recorded on micropillar surfaces treated with a second laser texturing step.

Moreover, Figure 9 presents the HTC values of micropillar surfaces with the additional laser texturing step at various heat flux levels. It is evident that all laser-textured surfaces demonstrated a notable enhancement in HTC compared to the untreated reference surface. Specifically, the highest HTCs were recorded at the critical heat flux or in the region of heat fluxes near the CHF. The surface with the hexagonal pattern and smaller micropillars exhibited the highest HTC, recording a value of 215 kW m^−2^ at CHF. Notably, surface H-20-LT displayed an HTC enhancement of 481% over the reference bare surface (REF). A possible explanation for the higher HTC values achieved on surfaces with smaller micropillars and additional laser texturing steps compared to surfaces with higher micropillars may be associated with the fact that taller micropillars can potentially extend the flow path of liquid and create greater flow resistance. This may prolong the detachment of the bubble from the surface leading to reduced HTC.

The distribution of micropillars also has an important role in the enhancement of the HTC values on surfaces with the additional laser treatment. It can be observed that the highest HTC at heat flux near and at the CHF, i.e., in a high heat flux region, was recorded for the surfaces with hexagonal micropillar patterns. This can be attributed to the moderate coalescence between bubbles resulting in vapor columns, forming distinct vapor–liquid pathways that were enabled on those surfaces due to the sufficient distance between the groups of micropillars. On the other hand, surfaces with circular and non-repetitive (Einstein) patterns exhibited lower enhancement of HTC, due to the more excessive coalescence of bubbles that was occurring due to insufficient distance between line-arranged micropillars for a circular pattern and especially insufficient distances between micropillars on the surface with a non-repetitive micropillar pattern. Therefore, it can be concluded that future studies on enhancing boiling performance on micropillar surfaces should focus on investigating the geometric optimization of the hexagonal micropillar patterns to determine the appropriate micropillar pitch that will result in high boiling performance. Furthermore, the stability of the micropillar surfaces tested after the second laser texturing step is demonstrated in Figures S13 and S14 in Supporting Information, Section S6.

### 3.4. Comparison of the Boiling Performance of Developed Surfaces

**Figure 10:**
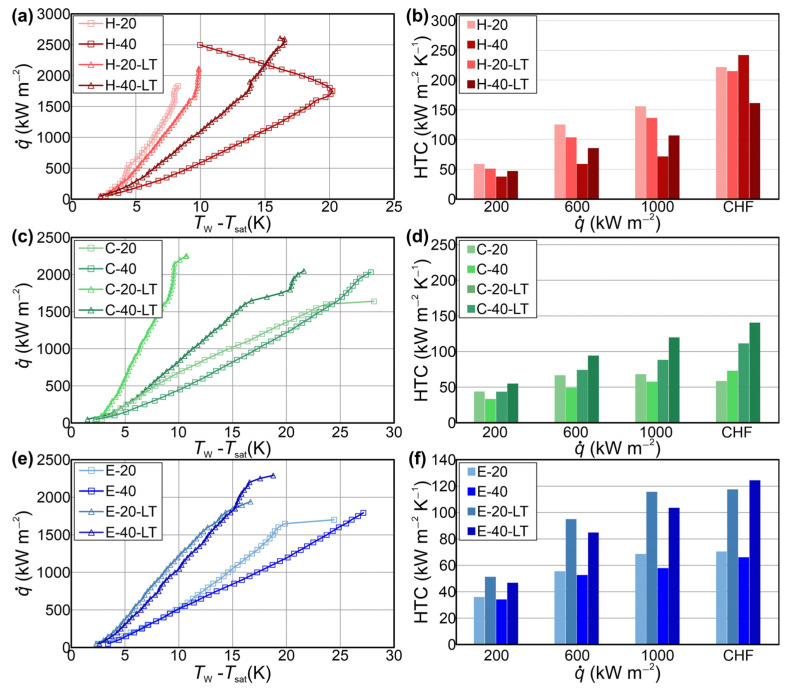
Comparison of boiling performance of tested micropillar samples: (**a**) boiling curves recorded on surfaces with the hexagonal boiling interface pattern, (**b**) HTC values recorded on surfaces with the hexagonal boiling interface pattern, (**c**) boiling curves recorded on surfaces with the circular boiling interface pattern, (**d**) HTC values recorded on surfaces with the circular boiling interface pattern, (**e**) boiling curves recorded on surfaces with the Einstein boiling interface pattern and (**f**) HTC values recorded on surfaces with the Einstein boiling interface pattern.

It was noted that surfaces with higher micropillars exhibited higher CHF values compared to the surfaces with lower micropillars, which can be mostly attributed to the increased surface area (see Supporting Information, Section S5). A comparison of the boiling performance of all tested micropillar samples is shown in Figure 10. The surfaces with higher micropillars exhibited increased CHF of up to 37% compared to their counterparts with smaller micropillars. Additionally, previous research indicates that the height of the trapped liquid in the intra-pillar liquid layer corresponds to the height of the pillar itself [61]. This alignment may offer an explanation for the enhanced CHF values observed on surfaces with higher pillars. The increased pillar height provides more space for the intra-pillar liquid layer, facilitating the retention of more trapped liquid during evaporation [61]. This, in conjunction with the wicking capabilities of the micropillar structure, results in a more effective delay of dry spot formation and higher CHF [61,62,63].

Surfaces with a second laser treatment exhibited higher enhancement of the CHF compared to their counterparts (tested after the chemical treatment in HCl). For example, the C-20-LT surface (having undergone the additional laser texturing step) exhibited a CHF enhancement of 38% compared to its counterpart (having only been etched in HCl). 

The increase in CHF values on samples with the additional laser texturing can also be attributed to the better wicking properties of the laser-textured surroundings compared to the chemically etched ones. This was confirmed by measurements of wickability in Figure S15, Supporting Information, Section S7. Moreover, the summary of the best-performing surfaces within this study is shown in Figure S16, Supporting Information, Section S8.

### 3.5. Evaluation of Heat Transfer Enhancement

**Figure 11:**
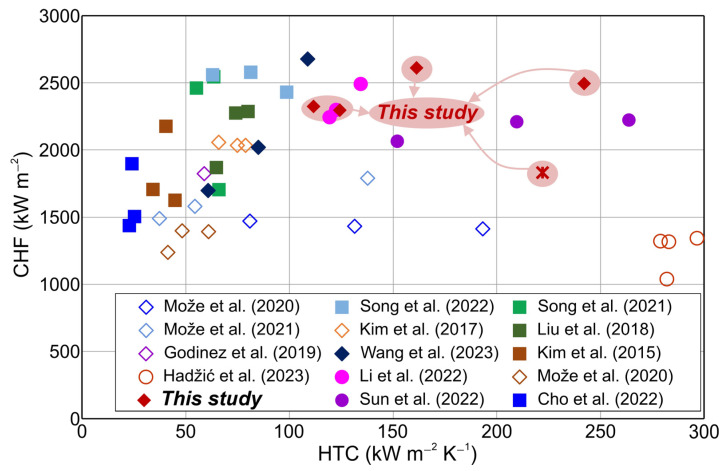
Comparison of the pool boiling performance of literature-reported surfaces [14,15,32,34,36,40,41,42,43,44,45,64,65,66] and the surfaces presented in this study.

To contextualize the attained boiling performance of the developed surfaces, a comparative analysis was conducted, comparing the CHF and HTC values obtained on the fabricated multiscale aluminum micropillar surfaces against relevant literature, as illustrated in Figure 11. It is noteworthy that most prior investigations involving micropillars focused on silicon and copper substrates, with minimal attention directed toward aluminum surfaces, whereas previous research mostly focused on structured surfaces without micropillars. Figure 10 shows a comparison of our data with previous findings obtained on aluminum, copper, and silicon substrates. Data were obtained on structured surfaces incorporating micropillars (filled symbols) and structured surfaces without micropillars (empty symbols). The data of the CHF and HTC shown in Figure 11 are summarized in Table S2, Supporting Information, Section S8.

The highest CHF was observed on the surface subjected to additional laser texturing and featuring higher micropillars (H-40-LT). Notably, the recorded CHF value ranks among the highest values reported in the existing literature on aluminum substrates. Furthermore, the micropillar surface characterized by a hexagonal pattern (H-40) exhibited the highest HTC, presenting values that are among the highest HTC values reported in the literature on aluminum surfaces. The sample H-40 demonstrated both a significantly increased CHF (2495 kW m^−2^) and a notably high HTC value (242 kW m^−2^ K^−1^). On the other hand, the sample H-40-LT exhibited the highest CHF but a lower HTC enhancement (161 kW m^−2^ K^−1^). Furthermore, the proposed fabrication processes are fast and cost-effective. The high boiling performance of these surfaces is very favorable for utilization in high-energy applications where high performance and high energy dissipation are needed.

## 4. Conclusions

This study presents a novel scalable approach to engineering micropillar surfaces with hierarchical micro-nanoscale structures on aluminum substrates for the simultaneous enhancement of critical heat flux and heat transfer coefficient during pool boiling. Three distinct micropillar patterns—hexagonal, circular, and Einstein—were evaluated by either testing the surfaces after the chemical etching or after an additional laser texturing step to modify the surrounding areas of the micropillars. The following conclusions were made based on the results of the performed experiments:

1. The surface with the hexagonal micropillar pattern of higher micropillars exhibited a CHF of 2495 kW m^−2^ and an HTC of 242 kW m^−2^ K^−1^. This configuration yielded enhancements of 126% and 554%, respectively, in comparison to the reference surface. Notably, the highest CHF value, reaching 2608 kW m^−2^, was observed on the hexagonal-patterned surface with additional laser texturing.
2. The pronounced improvement in CHF on micropillar surfaces compared to the untreated reference surface is ascribed to the formation of an intra-pillar liquid layer and increased capillary liquid inflow facilitated by the interpillar wickable structure that supplies the latter, leading to the delayed formation of dry spots.
3. The hexagonal micropillar pattern turned out to be the most effective, displaying moderate bubble coalescence, thereby improving the HTC. In high heat flux regions, the hexagonal pattern facilitated the generation of distinct vapor–liquid pathways, mitigating the formation of dryout spots and consequently yielding the highest CHF values in this study.
4. An exploration of two distinct micropillar heights revealed that higher micropillars result in a greater improvement in boiling heat transfer parameters, which is attributed to the heightened ability of taller micropillars to capture a greater intra-pillar liquid layer and facilitate improved liquid supply inflow toward the micropillars, leading to the delay of the formation of dry spots.
5. The presented study marks a meaningful foundation for efficient and scalable manufacturing of high-performing boiling interfaces on aluminum substrates, raising important future challenges of micropillar pattern optimization for achieving even higher simultaneous improvements in the critical heat flux and boiling heat transfer coefficient.

## Figures and Tables

**Table 1:** Standard measurement uncertainties of heat flux, heat transfer, and temperature superheat at different heat flux levels.

Heat Flux Level [kW m^−2^]	Surface Superheat [K]	Heat Flux [%]	Heat Transfer Coefficient [%]
200	0.7	9.4–10.9	11–12.1
800	1.1	4–15	8.8–13.2
2000	1.3	12–16	14–18.8

**Table 2:** List of surfaces fabricated and tested within the study.

Sample Name	Micropillar Average Height (µm)	Etching Time (Minutes)	Boiling Interface Pattern	Fabrication Step
REF	/	/	/	/
H-20	369 ± 15	20	Hexagonal	Laser texturing + Chemical etching
H-40	569 ± 28	40		
C-20	369 ± 15	20	Circular	
C-40	569 ± 28	40		
E-20	369 ± 15	20	Einstein	
E-40	569 ± 28	40		
H-20-LT	369 ± 15	20	Hexagonal	Laser texturing + Chemical etching + Additional laser texturing
H-40-LT	569 ± 28	40		
C-20-LT	369 ± 15	20	Circular	
C-40-LT	569 ± 28	40		
E-20-LT	369 ± 15	20	Einstein	
E-40-LT	569 ± 28	40		

## Data Availability

Data are available from the authors upon reasonable request.

## Supplementary Materials

The following supporting information can be downloaded at: https://www.mdpi.com/article/10.3390/nano14080667/s1, Figure S1: (a) Heating block and sample assembly, (b) sample dimensions including thermocouple locations. Figure S2: Graphical representation of laser texturing step utilized for fabrication of micropillar base. Figure S3: Graphical representation of the second laser texturing step. Figure S4: Pool boiling experimental setup. Figure S5: SEM analysis of aluminum surface after chemical etching with circular micropillar pattern. Figure S6: SEM analysis of aluminum surface after chemical etching with hexagonal micropillar pattern. Figure S7: SEM analysis of aluminum surface after chemical etching with Einstein micropillar pattern. Figure S8: SEM analysis of aluminum surface after the additional laser texturing step with circular micropillar pattern. Figure S9: SEM analysis of aluminum surface after the additional laser texturing step with hexagonal micropillar pattern. Figure S10: SEM analysis of aluminum surface after the additional laser texturing step with Einstein micropillar pattern. Table S1: Real-to-projected surface area ratio of the designed microchannel surface. Figure S11: Boiling performance of the micropillar aluminum surfaces (immediately after chemical etching) evaluated through boiling curves through the utilization of the actual surface area (refer to A) and projected surfaces area (refer to P) for: a) surfaces with smaller micropillars and b) surfaces with higher micropillars. Figure S12: Comparison of boiling performance of the micropillar aluminum surfaces (after additional laser texturing step) evaluated through boiling curves through the utilization of the actual surface area (refer to A) and projected surfaces area (refer to P) for: a) surfaces with smaller micropillars and b) surfaces with higher micropillars. Figure S13: Stability of boiling performance on surfaces with smaller micropillars: (a) sample E-20, immediately after chemical etching and (b) sample H-20-LT, after second laser texturing step. Figure S14: Stability of boiling performance on surfaces with smaller micropillars: (a) sample C-40, immediately after chemical etching and (b) sample E-40-LT, after second laser texturing step. Figure S15: Wicked liquid volume versus time for different surface treatments of the micropillar surrounding area. Figure S16: A summary of the key results of this study, plotted as HTC versus surface superheat. Table S2: Summarized data from literature. Refs. [67,68,69,70] are cited in the Supplementary Materials.
