# Supplementary material for: Aluminum Micropillar Surfaces with Hierarchical Micro- and Nanoscale Features for Enhancement of Boiling Heat Transfer Coefficient and Critical Heat Flux
Source: Nanomaterials (Basel). 2024 Apr 11;14(8):667. doi: 10.3390/nano14080667 (PMC11054976; doi:10.3390/nano14080667)
Supplement: Supplementary file 1 [file nanomaterials-14-00667-s001.zip › nanomaterials-2938738-supplementary.pdf]

Supplementary Information

Aluminum Micropillar Surfaces with Hierarchical Micro- and Nanoscale Features for Enhancement of Boiling Heat Transfer Coefficient and Critical Heat Flux

Armin Hadžić, Matic Može *, Matevž Zupančič and Iztok Golobič

Faculty of Mechanical Engineering, University of Ljubljana, Aškerčeva 6, 1000 Ljubljana, Slovenia; armin.hadzic@fs.uni-lj.si (A.H.); matevz.zupancic@fs.uni-lj.si (M.Z.); iztok.golobic@fs.uni-lj.si (I.G.)

* Correspondence: matic.moze@fs.uni-lj.si, Tel.: +386-1-4771-309

Table of Contents

S1. Sample fabrication process

Figure S1. a) Heating block and sample assembly, b) sample dimensions including thermocouple locations.

S2. Laser functionalization process

Figure S2. Graphical representation of laser texturing step utilized for fabrication of micropillar base.

Figure S3. Graphical representation of the second laser texturing step.

S3. Experimental setup

Figure S4. Pool boiling experimental setup.

S4. Surface analysis

Figure S5. SEM analysis of aluminum surface after chemical etching with circular micropillar pattern.

Figure S6. SEM analysis of aluminum surface after chemical etching with hexagonal micropillar pattern.

Figure S7. SEM analysis of aluminum surface after chemical etching with Einstein micropillar pattern.

Figure S8. SEM analysis of aluminum surface after the additional laser texturing step with circular micropillar pattern.

Figure S9. SEM analysis of aluminum surface after the additional laser texturing step with hexagonal micropillar pattern.

Figure S10. SEM analysis of aluminum surface after the additional laser texturing step with Einstein micropillar pattern.

S5. Effect of the actual surface area

Table S1. Real-to-projected surface area ratio of the designed microchannel surface.

Figure S11. Boiling performance of the micropillar aluminum surfaces (immediately after chemical etching) evaluated through boiling curves through the utilization of the actual surface area (refer to A) and projected surfaces area (refer to P) for: a) surfaces with smaller micropillars and b) surfaces with higher micropillars.

Figure S12. Comparison of boiling performance of the micropillar aluminum surfaces (after additional laser texturing step) evaluated through boiling curves through the utilization of the actual surface area (refer to A) and projected surfaces area (refer to P) for: a) surfaces with smaller micropillars and b) surfaces with higher micropillars.

S6. Stability of boiling performance

Figure S13. Stability of boiling performance on surfaces with smaller micropillars: a) sample E-20, immediately after chemical etching and b) sample H-20-LT, after second laser texturing step.

Figure S14. Stability of boiling performance on surfaces with smaller micropillars: a) sample C-40, immediately after chemical etching and b) sample E-40-LT, after second laser texturing step.

S7. Wicking of micropillar surrounding area

Figure S15. Wicked liquid volume versus time for different surface treatments of the micropillar surrounding area.

S8. Brief summary of the best-performing surfaces

Figure S16. A summary of the key results of this study, plotted as HTC versus surface superheat.

S9. Comparison with Literature-Reported Data

Table S2. Summarized data from literature.

S10. References

S1. Sample fabrication process

Schematic representation of the sample

A sample mounted on the copper heating block is shown in *Figure S1.* A copper heating block is used to supply the heat from the cartridge heaters to the sample. Three thermocouples at a distance of 5 mm from one another are used to record the axial spatial temperature gradient in the sample, which is then used to calculate the heat flux and extrapolate the surface temperature. The thermocouple closest to the boiling surface is positioned 6.5 mm from the top of the sample. This subassembly is then inserted through the lower flange into the boiling vessel. KRYPTONi- 8xTH DAQ device was used for collecting all temperature signals as raw voltages. The temperature of each cold junction is recorded internally and used to offset the measurements to obtain correct temperature readings. The calculation of temperatures based on offset voltages was performed utilizing NIST 9th-degree polynomial. Data from DAQ device are acquired using Dewesoft X3 software.

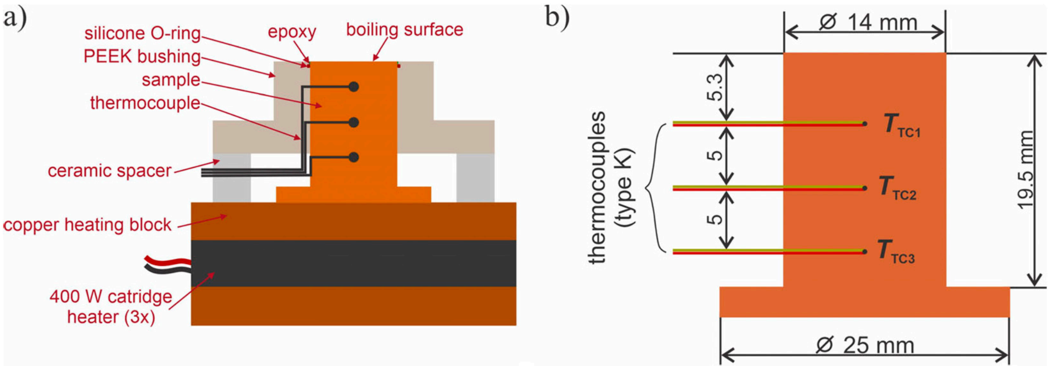

Figure S1. a) Heating block and sample assembly, b) sample dimensions including thermocouple locations.

S2. Laser functionalization process

The initial stage of laser texturing employed in the production of micropillar bases entailed the generation of microcavities and aluminum oxide layers. This laser processing phase involved the utilization of either five or ten laser passes to create bases characterized by varying thicknesses of the aluminum oxide layer. Specifically, one subset of samples underwent fabrication with five laser passes, resulting in the formation of a thinner aluminum oxide layer. Subsequently, these samples underwent chemical etching in HCl for 20 minutes. In contrast, a second subset of samples was manufactured using ten laser passes, yielding a thicker aluminum oxide layer. These samples then underwent chemical etching for a prolonged duration of 40 minutes. Consequently, two distinct sets of samples were obtained, each exhibiting micropillars of differing heights. A graphical representation of the structuring pattern scheme used for the fabrication of micropillars is shown in *Figure S2*.

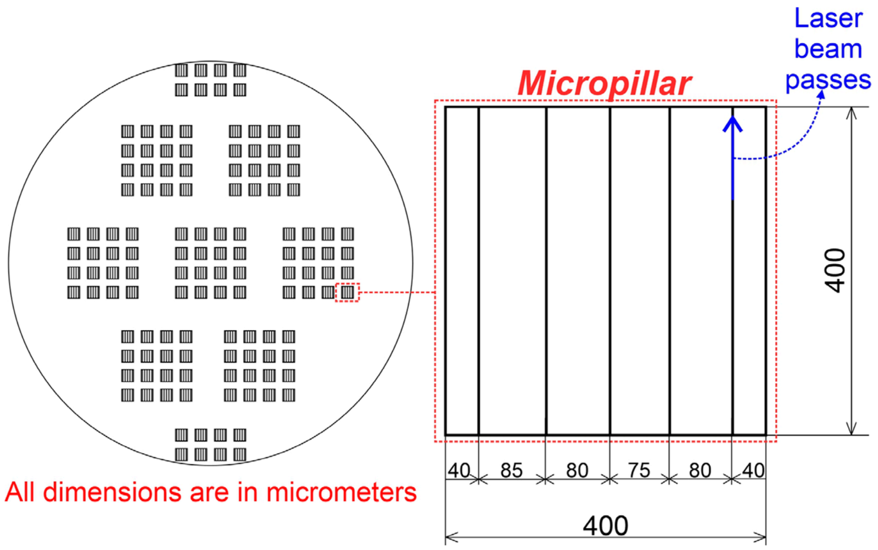

Figure S2. Graphical representation of laser texturing step utilized for fabrication of micropillar base.

The structuring scheme of the second laser texturing step used for the second set of micropillar aluminum surfaces is shown in *Figure S3*.

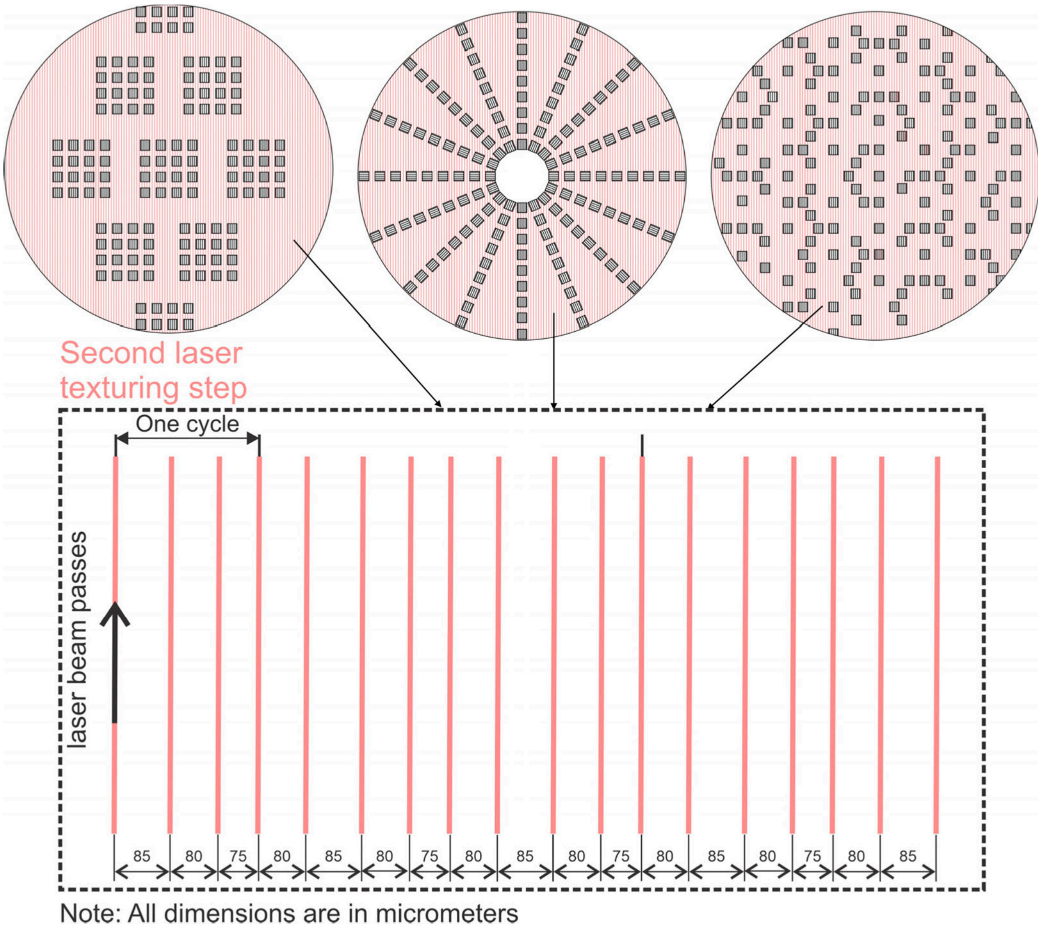

Figure S3. Graphical representation of the second laser texturing step.

S3. Experimental setup

A schematic depiction of the pool boiling experimental setup is shown in *Figure S4*. The boiling chamber is constructed of a glass cylinder with an inner diameter of 60 mm and placed between two stainless steel flanges. Produced vapor is condensed in a glass condenser, which is also vented to the atmosphere to prevent the pressure from rising above atmospheric pressure. Before each experiment, the working fluid (double distilled water) was preheated and degassed using an immersion heater controlled by a variable transformer. An immersion heater was also used to maintain the saturated state of the working fluid during the boiling experiment.

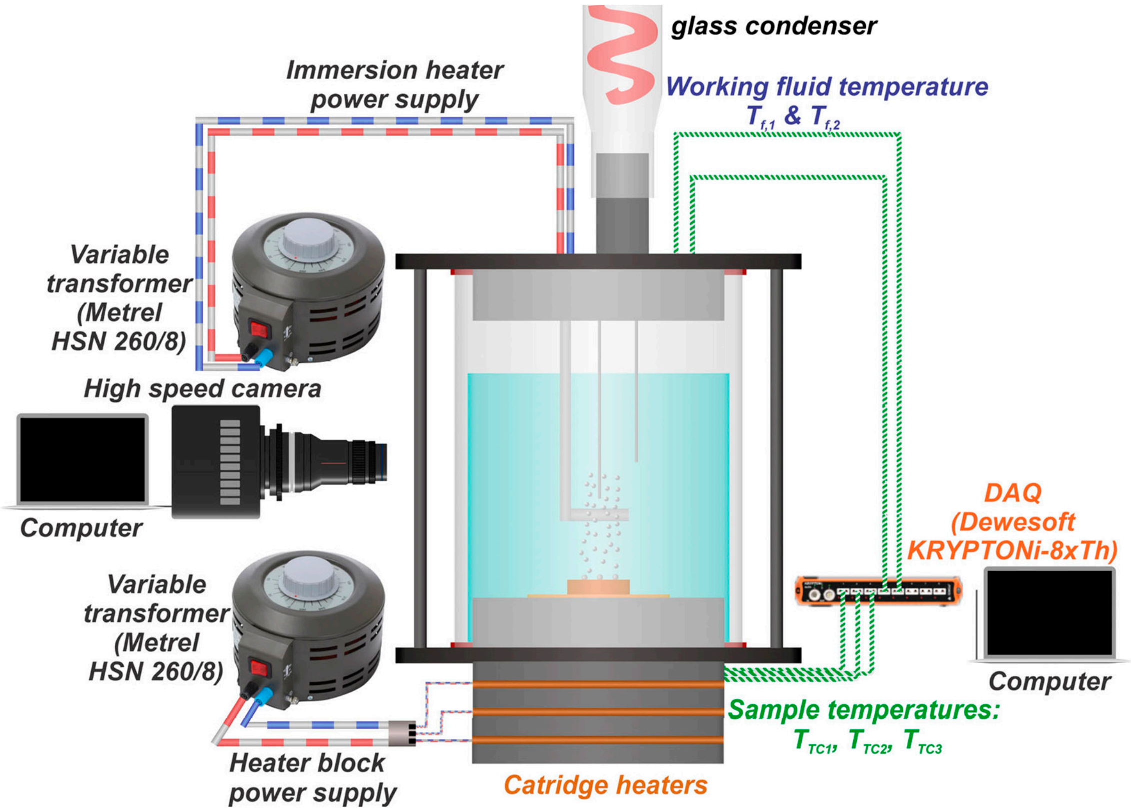

Figure S4. Pool boiling experimental setup.

The relevant heat transfer parameters were calculated using the temperatures measured in the sample and the boiling vessel by using a methodology that was utilized and thoroughly described in previous studies [1–3].

The contributing characteristics considered and the methodology used for the evaluation of the measurement uncertainty are in detail presented in our previous publication [4].

S4. Surface analysis

Figures S5-S7 show additional SEM images of the first set of fabricated surfaces with circular, hexagonal, and Einstein micropillar patterns. The first set of samples involves just surfaces that are fabricated utilizing the first step of laser texturing and chemical etching in HCl. SEM analysis was conducted of the aforementioned surfaces after boiling tests.

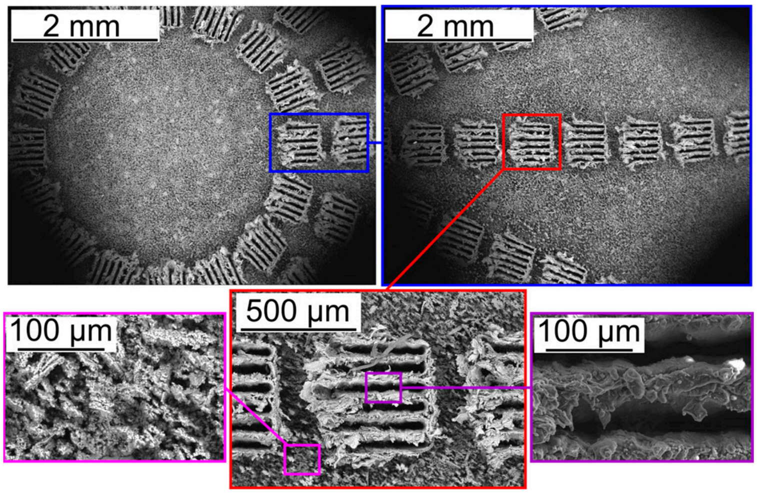

Figure S5. SEM analysis of aluminum surface after chemical etching with circular micropillar pattern.

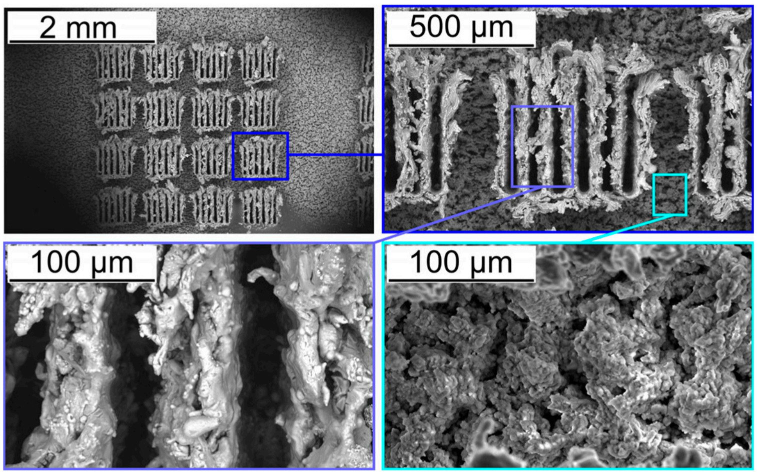

Figure S6. SEM analysis of aluminum surface after chemical etching with hexagonal micropillar pattern.

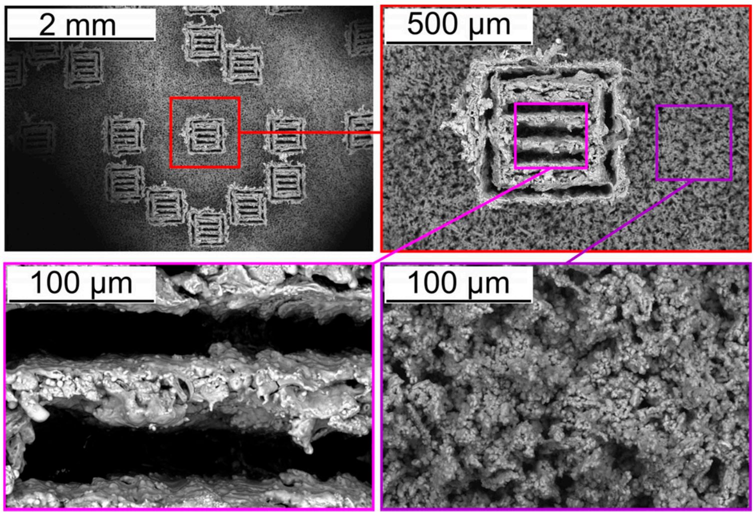

Figure S7. SEM analysis of aluminum surface after chemical etching with Einstein micropillar pattern.

Figures S8-S10 show additional SEM images of the second set of fabricated surfaces with circular, hexagonal, and Einstein micropillar patterns. The second set of samples involves surfaces that are fabricated utilizing the first step of laser texturing and chemical etching in HCl, and then undergo an additional (second) laser texturing step. SEM analysis of these surfaces was conducted after boiling tests.

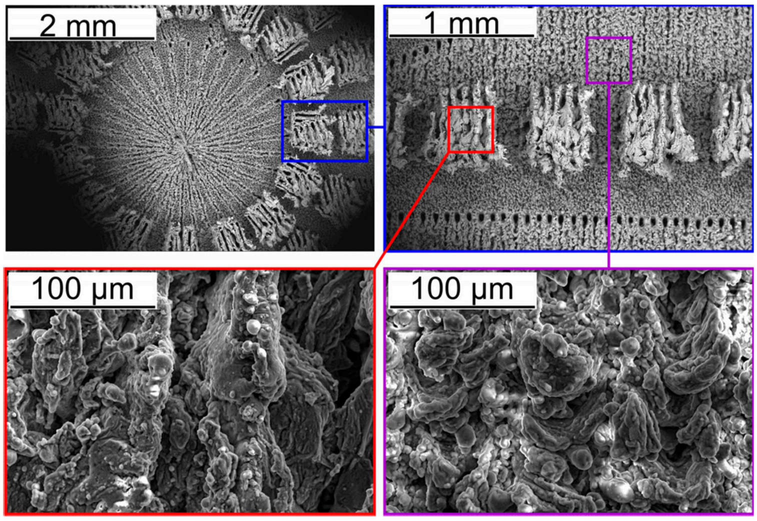

Figure S8. SEM analysis of aluminum surface after the additional laser texturing step with circular micropillar pattern.

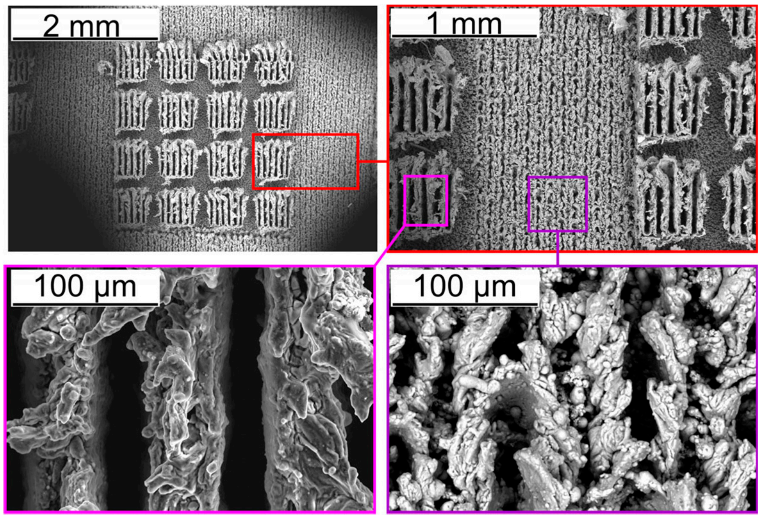

Figure S9. SEM analysis of aluminum surface after the additional laser texturing step with hexagonal micropillar pattern.

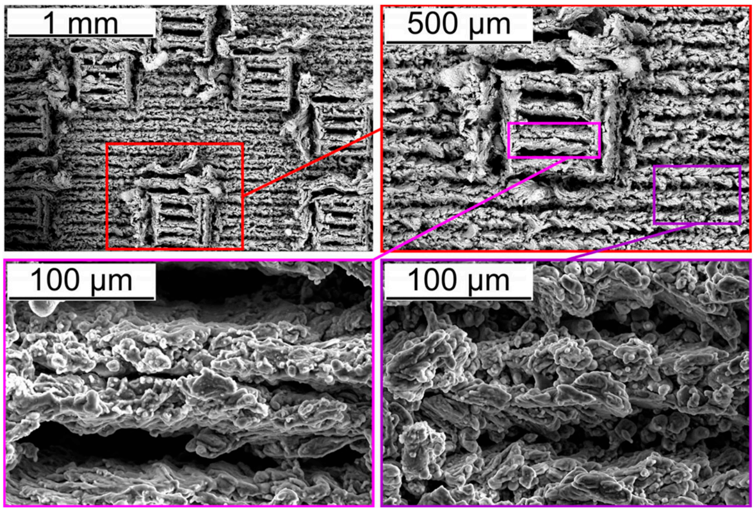

Figure S10. SEM analysis of aluminum surface after the additional laser texturing step with Einstein micropillar pattern.

S5. Effect of the actual surface area

Table S1 specifies the surface area increase in the tested surface due to the presence of micropillars.

Table S1. Real-to-projected surface area ratio of the designed microchannel surface.

Sample characteristics	H-20	H-40	C-20	C-40	E-20	E-40
Micropillar height (μm)	369	569	369	569	369	569
Number of micropillars	128	128	128	128	142	142
Micropillar width (μm) x length (μm)	400 x 400					
Real-to-projected surface area ratio	1.49	1.76	1.49	1.76	1.54	1.84

In *Figure S11*, a comparison is made between the boiling curves relying on the actual or projected surface area of the micropillar aluminum surfaces. In the boiling curve obtained based on the projected surface area, the area used for heat flux calculation is equal to the area of the reference sample, approximately 1.54 cm^{-2}. On the other hand, in the boiling curve obtained based on the actual surface area, the area used for heat flux calculations was the real area of the micropillar surfaces, and the real-to-projected surface area ratio is shown in *Table S1*. The results reveal that an increase in surface area has a huge effect that leads to the enhancement of boiling performance. It is observed that in both cases, smaller or higher micropillars, CHF enhancement is affected by surface area augmentation. It is observed that surface surfaces with hexagonal patterns still exhibited the CHF highest value, when heat fluxes were calculated using the actual s area, compared to two other patterns at both micropillar heights. Therefore, the CHF enhancement cannot be solely attributed to the augmented surface area, but the role of the micropillar pattern has a significant influence on CHF enhancement. Moreover, it is also observed that surfaces with Einstein pattern exhibited the largest argumentation of the surface area, and after calculation of the heat flux to the actual surface, those surfaces still exhibited low CHF values. Thus, the lowest enhancement of the CHF value with the Einstein pattern can be attributed to the small micropillar pitch on some parts of the sample which is highly suitable for the formation of small vapor films on such parts of the sample, causing eventual merging and possible earlier incipience of the boiling crisis.

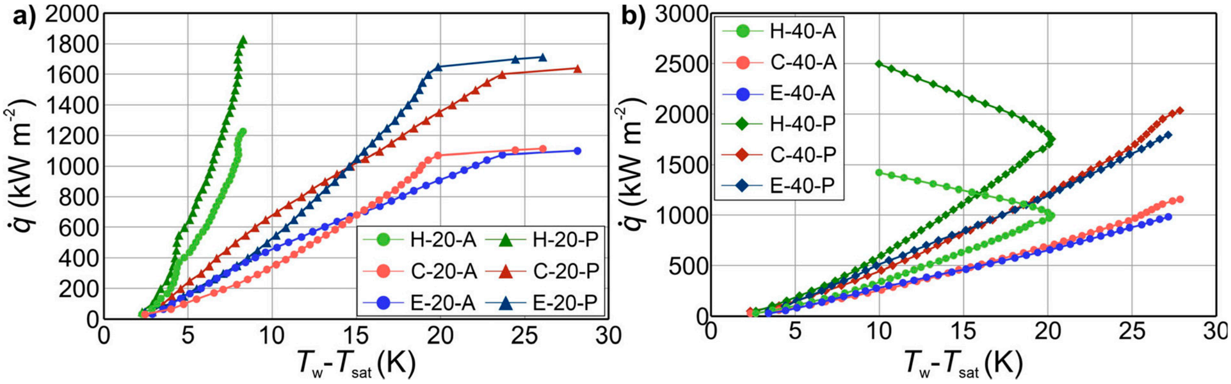

Figure S11. Boiling performance of the micropillar aluminum surfaces (immediately after chemical etching) evaluated through boiling curves through the utilization of the actual surface area (refer to A) and projected surfaces area (refer to P) for: a) surfaces with smaller micropillars and b) surfaces with higher micropillars.

Figure S12 shows the boiling curves relying on the actual or projected surface area of the micropillar aluminum surfaces (after an additional laser texturing step). Since the actual surface area of all micropillar surfaces is higher than the projected surface area, the boiling curves are shifted towards lower heat flux values. The results indicate that the surface area increase also has an impact on the enhancement of boiling heat transfer performance. It is observed that surfaces with higher micropillars exhibited higher CHF values, which can be attributed to the surface area augmentation.

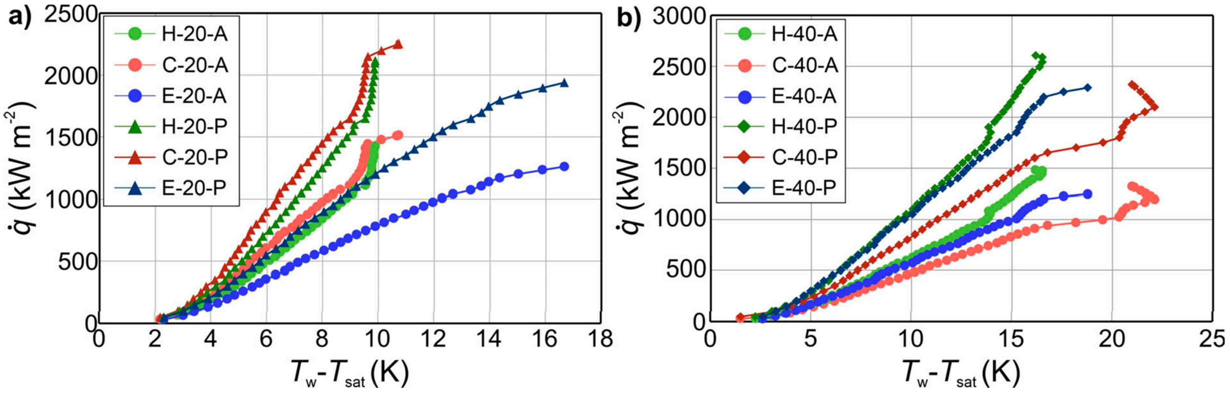

Figure S12. Comparison of boiling performance of the micropillar aluminum surfaces (after additional laser texturing step) evaluated through boiling curves through the utilization of the actual surface area (refer to A) and projected surfaces area (refer to P) for: a) surfaces with smaller micropillars and b) surfaces with higher micropillars.

S6. Stability of boiling performance

For instance, the stability of the boiling performance obtained on the E-20 surface with smaller micropillars, tested immediately after chemical etching is shown in *Figure S13a*, while the stability of the boiling performance obtained H-20-LT surface with smaller micropillars, tested after the second laser texturing step is shown in *Figure S13b*. For both samples it can be observed that boiling curves are shifted towards higher surface superheat during consecutive measurements. For sample E-20, the surface superheat was increased by 2.2 K, while for sample H-20-LT, the surface superheat increased by 1.4 K from the first to the third experimental run at a heat flux level of 800 kW m^{-2}. The measurement uncertainty of surface superheat was 1.1 K therefore the observed shift cannot be attributed to the measurement uncertainty. This shift can be attributed to the changes in the chemistry of the micropeak structure, where exposure to hot water causes a layer of boehmite to grow in the form of nanoneedles on aluminum samples [5–7]. The formation of a boehmite layer on aluminum surfaces after exposure to hot water is very well known and explained in the literature on chemically etched surfaces as well as laser-ablated surfaces [5–10]. The growth of boehmite nanoneedles can potentially increase the energy barrier for nucleation by reducing the number of open cavities. However, boehmite can also lead to an increase in heat transfer resistance within the porous structure and thus a reduction in boiling performance [5–7]. *Figure 4* in the manuscript shows the boehmite structures from on samples after the boiling test.

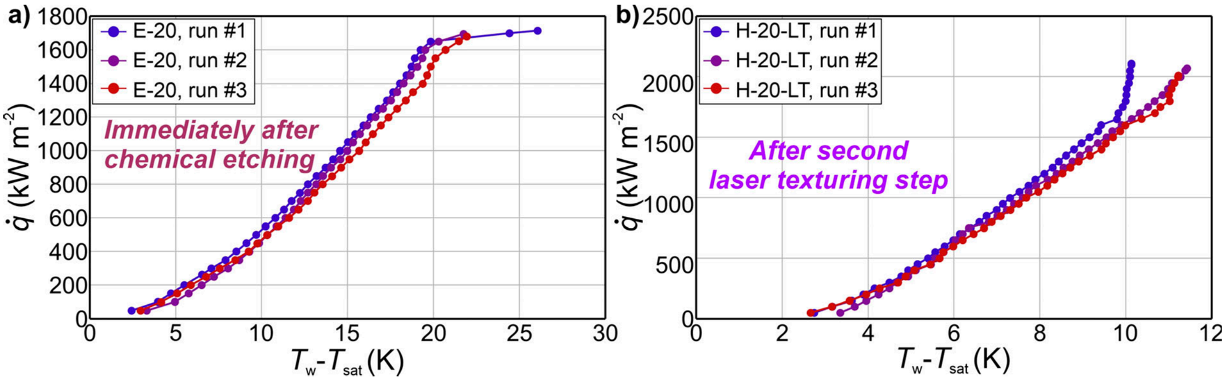

Figure S13. Stability of boiling performance on surfaces with smaller micropillars: a) sample E-20, immediately after chemical etching and b) sample H-20-LT, after second laser texturing step.

Moreover, the stability of the boiling performance obtained on the C-40 surface with smaller micropillars, tested immediately after chemical etching is shown in *Figure S14a*, while the stability of the boiling performance obtained E-40-LT surface with smaller micropillars, tested after the second laser texturing step is shown in *Figure S14b*. Surfaces with higher micropillars also showed a shift in boiling curves toward higher surface superheat. The surface superheat was increased by

2.6 K for sample C-40, while for sample E-40-LT, the surface superheat increased by 1.6 K at a heat flux at a heat flux level of 800 kW m^{-2} from the first to the third experimental run. The shift in the boiling curve can be attributed to the formation of the boehmite layer upon exposure to hot water, which leads to an increase in heat transfer resistance within the porous structure and thus a reduction in the boiling performance [5–7].

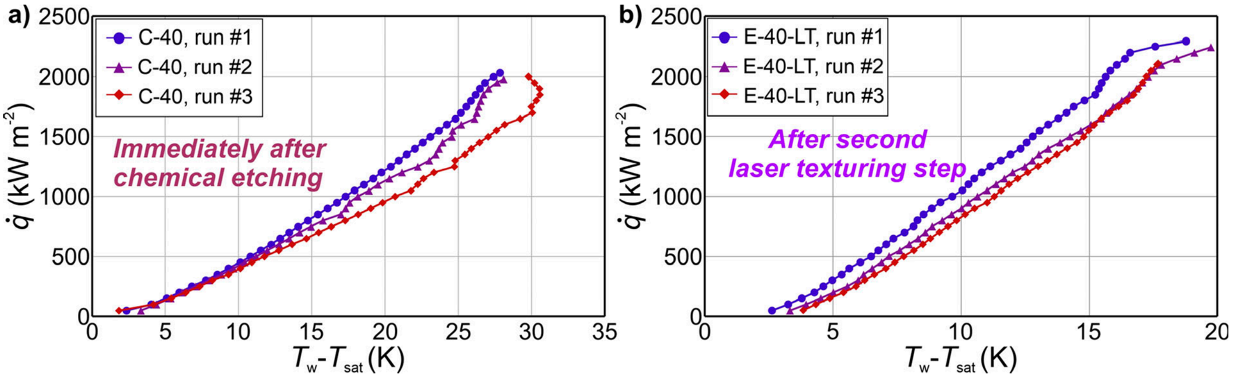

Figure S14. Stability of boiling performance on surfaces with smaller micropillars: a) sample C-40, immediately after chemical etching and b) sample E-40-LT, after second laser texturing step.

S7. Wicking of micropillar surrounding area

Wicking of the micropillar surrounding area was measured using the capillary tube method. The wicking measurements were performed on a custom experimental setup. We tested the wicking of the micropillar surrounding areas, by fabricating four separate aluminum samples. Two samples underwent chemical etching for 20 minutes and 40 minutes which is the same as with micropillar surrounding of the first type of surface. The third and fourth sample were prepared by a combination of chemical etching (20 and 40 minutes) and the additional laser texturing step. Three wicking tests were performed on each surface using double distilled water. The results of wicking evaluation are shown in *Figure S15*. It can be observed that surfaces with the additional laser texturing step wick a higher amount of liquid compared to the surfaces that only underwent chemical etching. The total wicked volume on the surface etched for 20 minutes was found to be ~5 μL after 13 s, while etching the sample for 40 minutes increased it to ~7.5 μL, corresponding to a 50% increase. Surfaces etched for 20 and 40 minutes and then treated with the additional laser texturing step wicked similar volumes of liquid, *i.e.*, ~8.87 μL after 13 s, an increase of 78% compared to the surface that was only etched for 20 minutes. Under the conditions adopted in this work, the micropillar surface surrounding areas fabricated with a combination of chemical etching and laser texturing is identified to be the best for preparing a highly wicking surface for increased liquid replenishment.

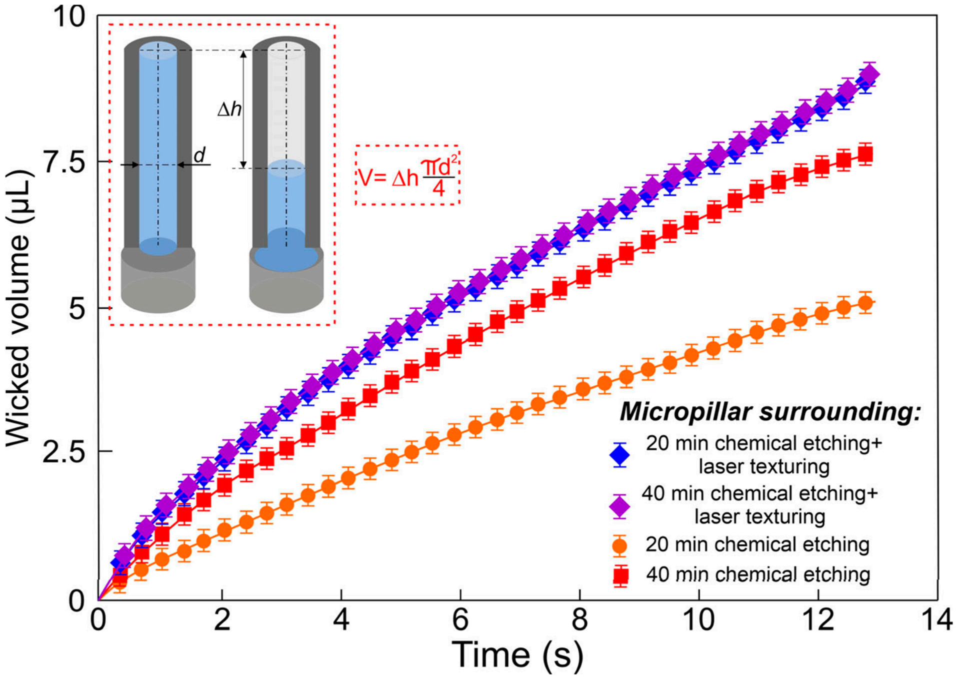

Figure S15. Wicked liquid volume versus time for different surface treatments of the micropillar surrounding area.

S8. Brief summary of the best-performing surfaces

Figure S16 presents a summary of the key results concerning HTC and surface superheat values. Our findings demonstrate that surfaces featuring hexagonal patterns and tested immediately after chemical etching or following additional laser texturing steps exhibited superior performance.

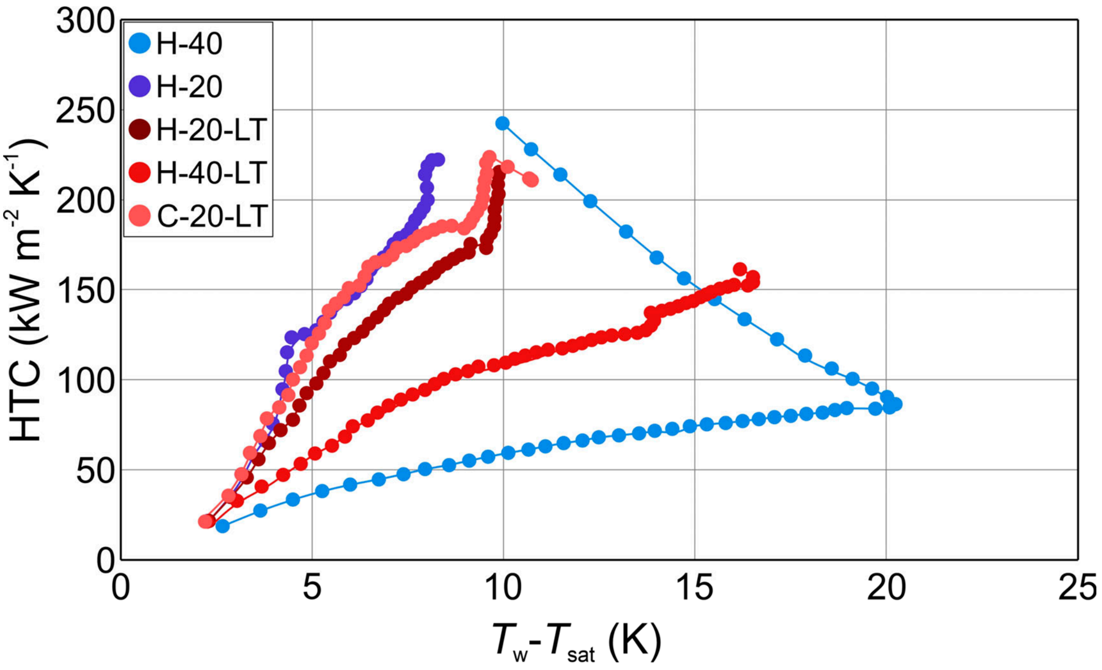

Figure S16. A summary of the key results of this study, plotted as HTC versus surface superheat.

Furthermore, it is evident that the highest HTC within a high heat flux region, specifically at CHF, was recorded on sample H-40. In the high heat flux region, surface H-40 exhibited the highest HTC value due to the activation of additional nucleation sites on top of the laser microstructures, not solely within the cavities. This phenomenon led to the occurrence of secondary boiling effects, resulting in a significant decrease in superheat within the high heat flux region.

Conversely, in a low heat flux region within the heat flux range up to 300 kW m^{-2}, surface H-20 demonstrated the best performance. For instance, at a heat flux of 200 kW m^{-2}, surface H-20 exhibited an HTC of 59 kW m^{-2} K^{-1}, which is 26% higher compared to its counterpart with larger micropillars.

Furthermore, in the high heat flux region, surfaces featuring hexagonal patterns tested after undergoing additional laser texturing steps exhibited lower HTC values compared to surfaces with hexagonal micropillar patterns tested immediately after chemical etching. Additionally, for instance, although surface H-40-LT exhibited a higher CHF value compared to surface H-40, the significantly lower HTC obtained on the surface with the additional laser texturing step led to the conclusion that surface H-40 represents the best-performing surface in this study.

S9. Comparison with Literature-Reported Data

The data used in Figure 11. are summarized in *Table S2*. *Table S2* presents data for the highest CHF values and the HTC values recorded at those CHFs in the literature. Additionally, the data recorded on best-performing surfaces from our study are also shown in *Table S2*.

Table S2. Summarized data from literature.

**Authors**	**CHF (kW m^{-2})**	**HTC (kW m^{-2} K^{-1})**	**Material**	**Surface structure**
Godinez et al. [7]	1820	59	Aluminum	Boehmite structure
Kim et al.[11]	2029	79	Aluminum	Boehmite structure
	2029	75		
	2052	66		
Može et al.[12]	1412	193	Aluminum	Laser textured structure
	1431	131		
	1470	81		
Može et al.[6]	1389	61	Aluminum	Laser-textured structure (Biphilic surfaces)
	1235	41		
	1394	48		
Može et al. [5]	1790	138	Aluminum	Boehmite structure
	1490	37		
	1580	54		
Wang et al.[13]	2674	109	Aluminum	Micropillar surface structure
	2017	85		
	169	61		
Song et al.[14]	2542	64	Silicon	Micropillar surface structure
	2459	55		
	1702	66		
Kim et al.[15]	2175	40	Silicon	Micropillar surface structure
	1703	34		
	1623	45		
Li et al.[16]	2490	134	Copper	Micropillar surface structure
	2299	123		
	2242	119		
Sun et al. [17]	2221	264	Copper	Micropillar surface structure
	2207	210		
	2062	152		
Cho et al.[18]	1433	23	Silicon	Micropillar surface structure
	1503	25		
	1894	24		

Liu et al. [19]	2280	79	Silicon	Micropillar surface structure
	2280	75		
	1862	64		
Song et al. [20]	2430	99	Silicon	Micropillar surface structure
	2577	81		
	2559	63		
Hadžić et al. [21]	1322	279	Copper	Laser-textured structure (Superbiphilic surface)
	1038	282		
	1317	283		
	1337	299		
This study	2495	242	Aluminum	Micropillar surface structure
	2609	161		
	2292	125		
	2323	112		
	1830	222		

S10. References

1. Može, M.; Senegačnik, M.; Gregorčič, P.; Hočevar, M.; Zupančič, M.; Golobič, I. Laser-Engineered Microcavity Surfaces with a Nanoscale Superhydrophobic Coating for Extreme Boiling Performance. *ACS Appl. Mater. Interfaces* **2020**, *12*, 24419–24431, doi:10.1021/acsami.0c01594.

2. Hadžić, A.; Može, M.; Arhar, K.; Zupančič, M.; Golobič, I. Effect of Nanoparticle Size and Concentration on Pool Boiling Heat Transfer with TiO2 Nanofluids on Laser-Textured Copper Surfaces. *Nanomaterials* **2022**, *12*, doi:10.3390/nano12152611.

3. Vajc, V.; Može, M.; Hadžić, A.; Šulc, R.; Golobič, I. Saturated and Subcooled Pool Boiling Heat Transfer in Mixtures of Water and Glycerin. *Exp. Heat Transf.* **2022**, *00*, 1–29, doi:10.1080/08916152.2022.2027574.

4. Može, M.; Zupančič, M.; Golobič, I. Investigation of the Scatter in Reported Pool Boiling CHF Measurements Including Analysis of Heat Flux and Measurement Uncertainty Evaluation Methodology. *Appl. Therm. Eng.* **2020**, *169*, doi:10.1016/j.applthermaleng.2020.114938.

5. Može, M.; Vajc, V.; Zupančič, M.; Šulc, R.; Golobič, I. Pool Boiling Performance of Water and Self-Rewetting Fluids on Hybrid Functionalized Aluminum Surfaces. *Processes* **2021**, *9*, doi:10.3390/pr9061058.

6. Može, M.; Zupančič, M.; Golobič, I. Pattern Geometry Optimization on Superbiphilic Aluminum Surfaces for Enhanced Pool Boiling Heat Transfer. *Int. J. Heat Mass Transf.* **2020**, *161*, doi:10.1016/j.ijheatmasstransfer.2020.120265.

7. Godinez, J.C.; Fadda, D.; Lee, J.; You, S.M. Development of a Stable Boehmite Layer on Aluminum Surfaces for Improved Pool Boiling Heat Transfer in Water. *Appl. Therm. Eng.* **2019**, *156*, 541–549, doi:10.1016/j.applthermaleng.2019.04.065.

8. Fang, R.; Zhang, X.; Zheng, J.; Pan, Z.; Yang, C.; Deng, L.; Li, R.; Lai, C.; Yan, W.; Maisotsenko, V.S.; et al. Superwicking Functionality of Femtosecond Laser Textured Aluminum at High Temperatures. *Nanomaterials* **2021**, *11*, 1–19, doi:10.3390/nano11112964.

9. Tran, N.G.; Chun, D.M. Green Manufacturing of Extreme Wettability Contrast Surfaces with Superhydrophilic and Superhydrophobic Patterns on Aluminum. *J. Mater. Process. Technol.* **2021**, *297*, 117245, doi:10.1016/j.jmatprotec.2021.117245.

10. Freitas, E.; Pontes, P.; Cautela, R.; Bahadur, V.; Miranda, J.; Ribeiro, A.P.C.; Souza, R.R.; Oliveira, J.D.; Copetti, J.B.; Lima, R.; et al. Article Pool Boiling of Nanofluids on Biphilic Surfaces: An Experimental and Numerical Study. *Nanomaterials* **2021**, *11*, 1–23, doi:10.3390/nano11010125.

11. Kim, J.; Jun, S.; Lee, J.; Godinez, J.; You, S.M. Effect of Surface Roughness on Pool Boiling Heat Transfer of Water on a Superhydrophilic Aluminum Surface. *J. Heat Transfer* **2017**, *139*, 1–9, doi:10.1115/1.4036599.

12. Može, M.; Senegačnik, M.; Gregorčič, P.; Hočevar, M.; Zupančič, M.; Golobič, I. Laser-Engineered Microcavity Surfaces with a Nanoscale Superhydrophobic Coating for Extreme Boiling Performance. *ACS Appl. Mater. Interfaces* **2020**, *12*, 24419–24431, doi:10.1021/acsami.0c01594.

13. Wang, X.; Xu, J.; Jiang, H.; Liu, Y.; Li, X.; Shan, D.; Guo, B. Achieving Robust and Enhanced Pool Boiling Heat Transfer Using Micro–Nano Multiscale Structures. *Appl. Therm. Eng.* **2023**, *227*, 120441, doi:10.1016/j.applthermaleng.2023.120441.

14. Song, Y.; Gong, S.; Vaartstra, G.; Wang, E.N. Microtube Surfaces for the Simultaneous Enhancement of Efficiency and Critical Heat Flux during Pool Boiling. *ACS Appl. Mater. Interfaces* **2021**, *13*, 12629–12635, doi:10.1021/acsami.1c00750.

15. Kim, S.H.; Lee, G.C.; Kang, J.Y.; Moriyama, K.; Kim, M.H.; Park, H.S. Boiling Heat Transfer and Critical Heat Flux Evaluation of the Pool Boiling on Micro Structured Surface. *Int. J. Heat Mass Transf.* **2015**, *91*, 1140–1147, doi:10.1016/j.ijheatmasstransfer.2015.07.120.

16. Li, Q.; Zhao, J.; Sun, X.; Liu, B. Experimental Investigation of Pool Boiling Heat Transfer on Pillar-Structured Surfaces with Different Wettability Patterns. *Appl. Therm. Eng.* **2022**, *215*, 118924, doi:10.1016/j.applthermaleng.2022.118924.

17. Sun, X.Z.; Li, Q.; Li, W.X.; Wen, Z.X.; Liu, B. Enhanced Pool Boiling on Microstructured Surfaces with Spatially-Controlled Mixed Wettability. *Int. J. Heat Mass Transf.* **2022**, *183*, doi:10.1016/j.ijheatmasstransfer.2021.122164.

18. Cho, H.R.; Park, S.C.; Kim, D.; Joo, H.M.; Yu, D.I. Experimental Study on Pool Boiling on Hydrophilic Micro/Nanotextured Surfaces with Hydrophobic Patterns. *Energies* **2021**, *14*, 1–13, doi:10.3390/en14227543.

19. Liu, Y.; Lu, M.C.; Xu, D. The Suppression Effect of Easy-to-Activate Nucleation Sites on the Critical Heat Flux in Pool Boiling. *Int. J. Therm. Sci.* **2018**, *129*, 231–237, doi:10.1016/j.ijthermalsci.2018.03.007.

20. Song, Y.; Díaz-Marín, C.D.; Zhang, L.; Cha, H.; Zhao, Y.; Wang, E.N. Three-Tier Hierarchical Structures for Extreme Pool Boiling Heat Transfer Performance. *Adv. Mater.* **2022**, *34*, doi:10.1002/adma.202200899.

21. Hadžić, A.; Može, M.; Zupančič, M.; Golobič, I. Superbiphilic Laser-Microengineered Surfaces with A Self-Assembled Monolayer Coating for Exceptional Boiling Performance. *Adv. Funct. Mater.* **2023**, *2310662*, 1–14, doi:10.1002/adfm.202310662.
